# Differences in Somatic Mutations Between Early-Onset and Late-Onset Colorectal Cancer: A Systematic Review and Meta-Analysis

**DOI:** 10.3390/cancers18193225

**Published:** 2026-10-07

**Authors:** Hui Lionel Raphael Chen, Jun Yan Ian Wee, Jun Kiat Thaddaeus Tan, Wei Ming Aaron Seah, Anders Jacobsen Skanderup, Chin-Ann Johnny Ong, Han Chong Toh, Kwong-Wei Emile Tan, Bee Huat Iain Tan

**Affiliations:** 1 Department of Colorectal Surgery, Singapore General Hospital, Singapore 169608, Singapore; 2 Health Services Research Unit, Singapore General Hospital, Singapore 169608, Singapore; 3 Genome Institute of Singapore (GIS), Agency for Science, Technology and Research (A*STAR), Singapore 138672, Singapore; 4 Department of Sarcoma, Peritoneal and Rare Tumors (SPRinT), Division of Surgery and Surgical Oncology, National Cancer Centre Singapore, Singapore 168583, Singapore; 5 Division of Medical Oncology, National Cancer Centre Singapore, Singapore 168583, Singapore

**Keywords:** early-onset colorectal cancer, somatic mutations, driver genes

## Abstract

Background: Early-onset colorectal cancer (EOCRC, diagnosed <50 years) is increasing globally and presents with advanced clinicopathological features, yet its mutational landscape compared to late-onset CRC (LOCRC) remains poorly defined. Methods: We systematically searched PubMed and Embase (January 2016–December 2025) for studies comparing somatic mutations between EOCRC and LOCRC. Two reviewers independently screened 1880 records; 43 studies met inclusion criteria. Two public datasets (TCGA, China PanCancer) were also included. Meta-analyses were conducted using random-effects models with Review Manager. Subgroup analysis was restricted to microsatellite-stable (MSS) tumors. Meta-regression evaluated study-level differences in tumor sidedness and stage distribution as sources of heterogeneity. The primary outcome was odds ratios (OR) for mutations in 10 driver genes. Results: Among 154,982 participants, 33,937 (21.9%) had EOCRC. In the overall analysis, EOCRC showed higher frequencies of *TP53* (OR 1.13), *SMAD4* (OR 1.15), and *PTEN* mutations (OR 1.77), and lower frequencies of *APC* (OR 0.73), *KRAS* (OR 0.94), and *BRAF* mutations (OR 0.69). In MSS-restricted analysis, differences persisted for *TP53*, *SMAD4*, *APC*, and *KRAS*, whereas *PTEN* and *BRAF* differences became non-significant. Additionally, *NRAS* (OR 0.74) and *PIK3CA* mutations (OR 0.89) were less frequent in MSS-EOCRC. Meta-regression showed that study-level differences in left-sided tumor distribution were associated with lower relative BRAF mutation frequency in EOCRC (β = −5.08, 95% CI −7.66 to −2.49, *p* < 0.001). Conclusions: EOCRC exhibits a different mutational profile with higher *TP53* and *SMAD4* and lower *APC* and *KRAS* mutations. These findings highlight the need for further research into alternative mechanisms of CRC carcinogenesis among younger adults.

## Simple Summary

Early-onset colorectal cancer (EOCRC), diagnosed before age 50, is increasing globally, but its mutational differences from late-onset colorectal cancer (LOCRC) are not fully understood. This meta-analysis analyzed 43 studies and two public datasets, including 154,982 participants. Results showed that EOCRC tumors more often had mutations in *TP53, SMAD4*, and *PTEN* genes, but fewer mutations in *APC*, *KRAS*, and *BRAF* compared to LOCRC. When focusing only on microsatellite-stable tumors, differences in *TP53*, *SMAD4*, *APC*, and *KRAS* remained, while *PTEN* and *BRAF* differences disappeared. The lower *BRAF* rate in younger patients was linked to having more left-sided tumors. These findings highlight the need for further research into alternative mechanisms of CRC carcinogenesis in younger adults.

## 1. Introduction

Colorectal cancer (CRC) represents a significant global health challenge and ranks as the third leading cause of cancer-related mortality [1,2]. Although the increased uptake of CRC screening has contributed to the decreasing incidence and mortality rates of CRC among older populations, the incidence of early-onset colorectal cancer (EOCRC)—defined as CRC diagnosed in individuals below 50 years of age—has been consistently rising [3,4,5]. Longitudinal studies in the U.S. and Australia confirm persistent annual increases in incidence of 1 to 2% among adults aged 40–49 since the 1990s, a trend now also documented in several Asian countries, and is likely to evolve into a significant public health challenge in the upcoming decades [6,7,8,9,10,11].

Unlike late-onset colorectal cancer (LOCRC), EOCRC is not typically detected through CRC screening. EOCRC patients frequently present at a more advanced stage with more aggressive pathological characteristics such as poorly differentiated, signet-ring, or mucinous histology, as well as lymphovascular or perineural invasion, all of which correlate with a poorer prognosis [12,13,14]. These unique histopathological features suggest that EOCRC may arise through alternative biological mechanisms. Comparative genomic studies of EOCRC and LOCRC have revealed subtle differences, but the findings remain inconsistent. One of the most notable differences is the higher prevalence of microsatellite instability-high (MSI-H) tumors in EOCRC. MSI-H is observed in 10-15% of EOCRC cases compared to 2–7% of LOCRC [15].

A comprehensive study demonstrated that while most gene alteration frequencies were similar, *TP53* and *CTNNB1* mutations were more prevalent in EOCRC, whereas alterations in *APC*, *KRAS*, *BRAF*, and *PIK3CA* were comparatively less common [16]. Conversely, another study identified only marginal differences in the *TP53* and RTK-RAS pathways. After controlling for tumor location, there were no statistically significant differences at either the gene or pathway level [17]. Another study observed no difference in Wnt signaling pathway alterations, but TGF-b pathway mutations were more prevalent in LOCRC [18]. While there have been several large-scale observational studies that have compared the mutational profiles between EOCRC and LOCRC, the results have been conflicting, and the global molecular landscape of EOCRC remains unclear. Discrepancies in results may arise from potential confounders. MSI-H tumors are more prevalent in EOCRC compared to LOCRC and are characterized by a hypermutator phenotype and a distinct mutational profile, such as a higher frequency of *BRAF* mutations [15]. Additionally, EOCRC and LOCRC differ in tumor anatomical distribution, with a higher proportion of left-sided and rectal tumors in EOCRC [13]. As tumor sidedness is associated with distinct mutational profiles, differences in anatomical distribution in EOCRC and LOCRC may also confound the mutational differences observed.

Therefore, this systematic review and meta-analysis aims to consolidate the current evidence contrasting the differences in somatic gene mutations between EOCRC and LOCRC to gain further biological insights and inform treatment strategies. To evaluate age-specific mutational differences, a subgroup analysis restricted to MSS tumors was performed to remove the confounding effect of MSI tumors. Finally, meta-regression was also performed to evaluate study-level differences in tumor sidedness and stage distribution, which may contribute to mutational disparities in both age groups.

## 2. Methods

The systematic review was conducted in accordance with the PRISMA checklist and registered in PROSPERO with the number CRD420261320686.

### 2.1. Search Strategy and Study Selection

A literature search of PubMed and Embase was performed and limited to peer-reviewed, original research articles published in English within the last 10 years (from 1 January 2016 to 31 December 2025), with keywords related to colorectal cancer, early age of onset, and somatic mutational profiling (Supplementary Table S1). Two reviewers independently screened titles or abstracts and full texts, with disagreements resolved by a third reviewer.

### 2.2. Inclusion and Exclusion Criteria

Articles that compared the somatic mutations in EOCRC (age of diagnosis < 50 or ≤50 years) and LOCRC (age of diagnosis ≥ 50 years or >50 years) were included. Studies were excluded if there was no direct age-group comparison, or were focused on hereditary CRC syndromes or lacked analyzable mutational data. To avoid duplicate data and ensure the independence of samples, studies were also excluded if they overlapped with a previously published cohort in sample population or reported biomarkers, and only the most comprehensive study was retained for analysis. Additionally, we excluded studies that derived their results from large, publicly available datasets (e.g., TCGA) to prevent overlap in study populations. Supplementary Table S2 contains a list of studies excluded due to overlapping cohorts. To ensure that these cohorts were represented in the meta-analysis, we included the mutational data downloaded directly from cBioPortal [19] for two cohorts: TCGA [20] and China PanCancer [21]. Quality assessment of each study was assessed with Joanna Briggs Institute Critical Appraisal Tools for cohort studies, cross-sectional studies, randomized controlled trials and case–control studies [22]. Risk of bias was graded and separated into 3 categories: low risk, more than 75% of checklist items included; moderate risk, 50% to 75% of checklist items; and high risk, less than 50% of checklist items.

### 2.3. Outcomes of Interest

Data on study characteristics and clinicopathological features (tumor location, staging, grade and microsatellite status) were extracted from the included articles. A curated list of 10 common genes associated with CRC carcinogenesis (*APC*, *TP53*, *KRAS*, *PIK3CA*, *SMAD4*, *FBXW7*, *BRAF*, *NRAS*, *PTEN*, and *ATM*) from the AACR-GENIE database [23] was used to compare the mutational landscape between EOCRC and LOCRC. The denominator for each gene of interest includes patients whose tumors were sequenced for that gene. Studies were excluded from a gene analysis if the gene was not included in the sequencing panel.

### 2.4. Statistical Analysis

The primary summary measure was the odds ratio (OR) with corresponding 95% confidence intervals (CIs), comparing the prevalence of each mutation between EOCRC and LOCRC groups.

Meta-analyses were performed using Review Manager (RevMan 5). Pooled estimates were calculated using the inverse-variance method under a random-effects model to account for anticipated between-study heterogeneity arising from differences in study populations, sequencing methods, and tumor characteristics. Between-study variance (τ^2^) was estimated using the restricted maximum likelihood estimator (REML).

Statistical heterogeneity was assessed using Cochran’s Q test and quantified using the I^2^ statistic, with larger values indicating increasing heterogeneity. Summary effect estimates were reported with 95% Wald-type confidence intervals, and statistical significance was defined as a two-sided *p*-value < 0.05.

MSI-H colorectal cancers are characterized by a hypermutated phenotype and elevated tumor mutational burden, which confound comparisons of individual driver gene mutations. A separate analysis restricted to patients with microsatellite-stable (MSS) tumors was performed.

To explore potential sources of heterogeneity in gene mutation differences between EOCRC and LOCRC, meta-regression analyses were performed for genes with significant heterogeneity defined as I^2^ > 50%.

Study-level clinical and pathological characteristics were evaluated as potential moderators. These were defined by the difference in prevalence between EOCRC and LOCRC within each study (EOCRC minus LOCRC), including:

- proportion of left-sided tumors
- proportion of stage III–IV disease

This approach allowed assessment of whether differences in clinical composition between the two populations could explain variation in reported mutation effect sizes across studies.

Univariable meta-regression models were first fitted separately for each moderator, followed by multivariable models. Random-effects meta-regression models were fitted using REML estimation of the between-study variance (τ^2^). Moderator significance was assessed using the Wald test, and the proportion of between-study heterogeneity explained was quantified using R^2^. Residual heterogeneity was quantified using τ^2^ and I^2^ statistics and tested using the residual Cochran Q statistic. Given the limited number of studies available for some genes, multivariable models were restricted to a small number of moderators to minimize overfitting. Meta-regression analyses were performed using Stata (version 18.5; StataCorp, College Station, TX, USA).

Publication bias and small-study effects were assessed by visual inspection of funnel plots. For meta-analyses, including at least 10 studies, funnel plot asymmetry was additionally assessed using Egger’s regression test. Formal tests for funnel plot asymmetry were not performed for analyses containing fewer than 10 studies because of their limited statistical power. Sensitivity analyses excluding studies with total sample sizes < 100 were performed to assess the robustness of the pooled estimates to the inclusion of small studies. To address the potential impact of heterogeneous age cutoffs across included studies, a sensitivity analysis was performed by restricting to studies that used the definition of EOCRC as age < 50 years and LOCRC as age ≥ 50 years.

## 3. Results

**Figure 1:**
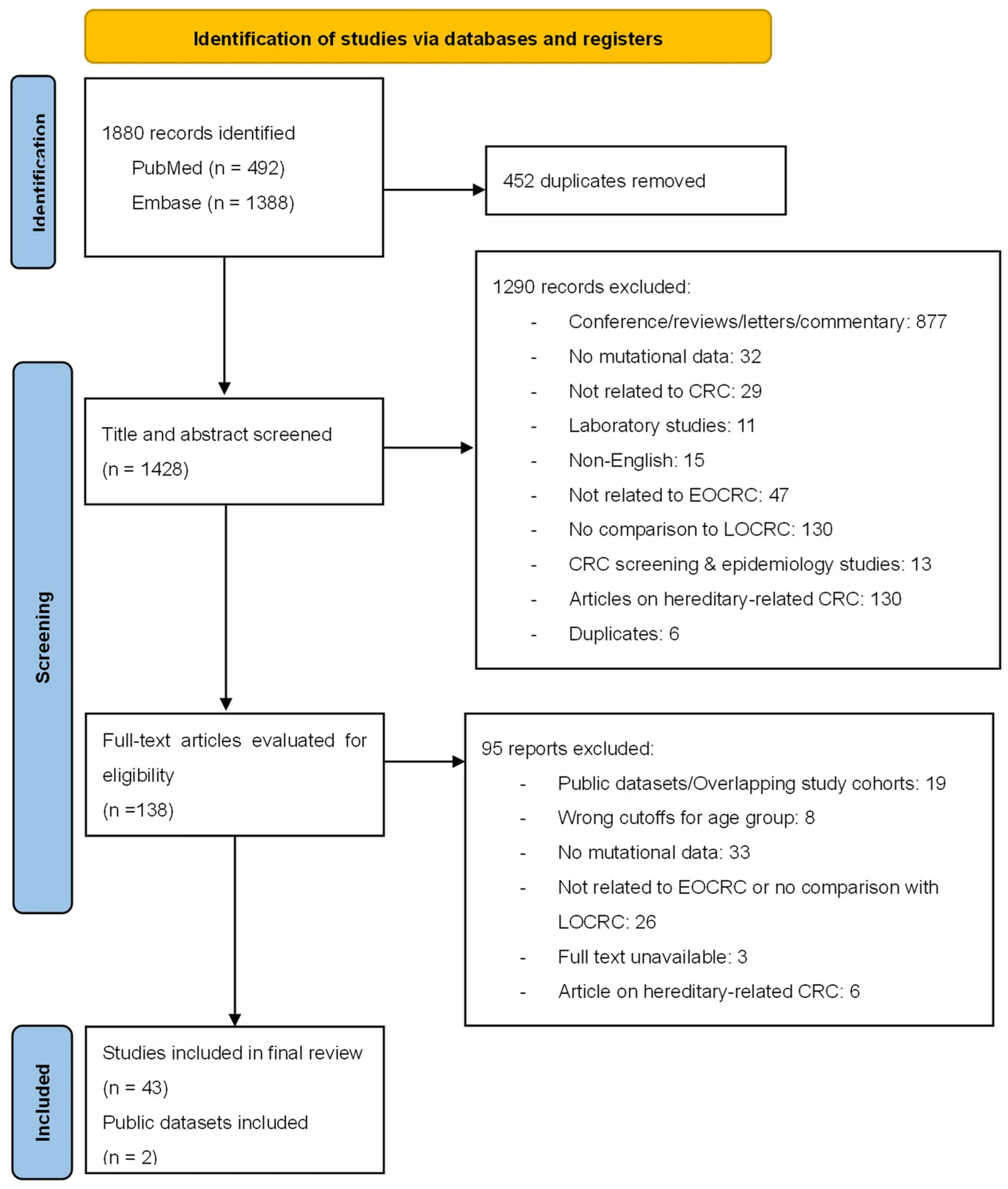
PRISMA Chart.

A total of 1880 papers were identified from PubMed and Embase, and 452 duplicates were removed. After initial screening, 138 studies were selected for full-text review, of which 43 studies and 2 public cohorts were included in the meta-analysis (Figure 1) [14,20,21,24,25,26,27,28,29,30,31,32,33,34,35,36,37,38,39,40,41,42,43,44,45,46,47,48,49,50,51,52,53,54,55,56,57,58,59,60,61,62,63,64,65].

### 3.1. Study Characteristics

This study included 154,982 participants with mutational data; 33,937 (21.9%) were EOCRC. Supplementary Table S3 summarizes the details of the included studies. Supplementary Tables S4–S6 summarize the quality assessment of the studies included.

### 3.2. Clinicopathological Features

The clinicopathological characteristics of EOCRC and LOCRC are summarized descriptively in Table 1. Based on aggregated data from the included studies, EOCRC patients had a higher proportion of stage III–IV disease at diagnosis compared with LOCRC patients (70.7% vs. 61.7%), as well as higher proportions of left-sided or rectal cancers (61.3% vs. 56.4%), poorly differentiated tumors (21.0% vs. 14.0%), and MSI tumors (13.1% vs. 9.0%).

### 3.3. Common Biomarker Mutations

**Figure 2:**
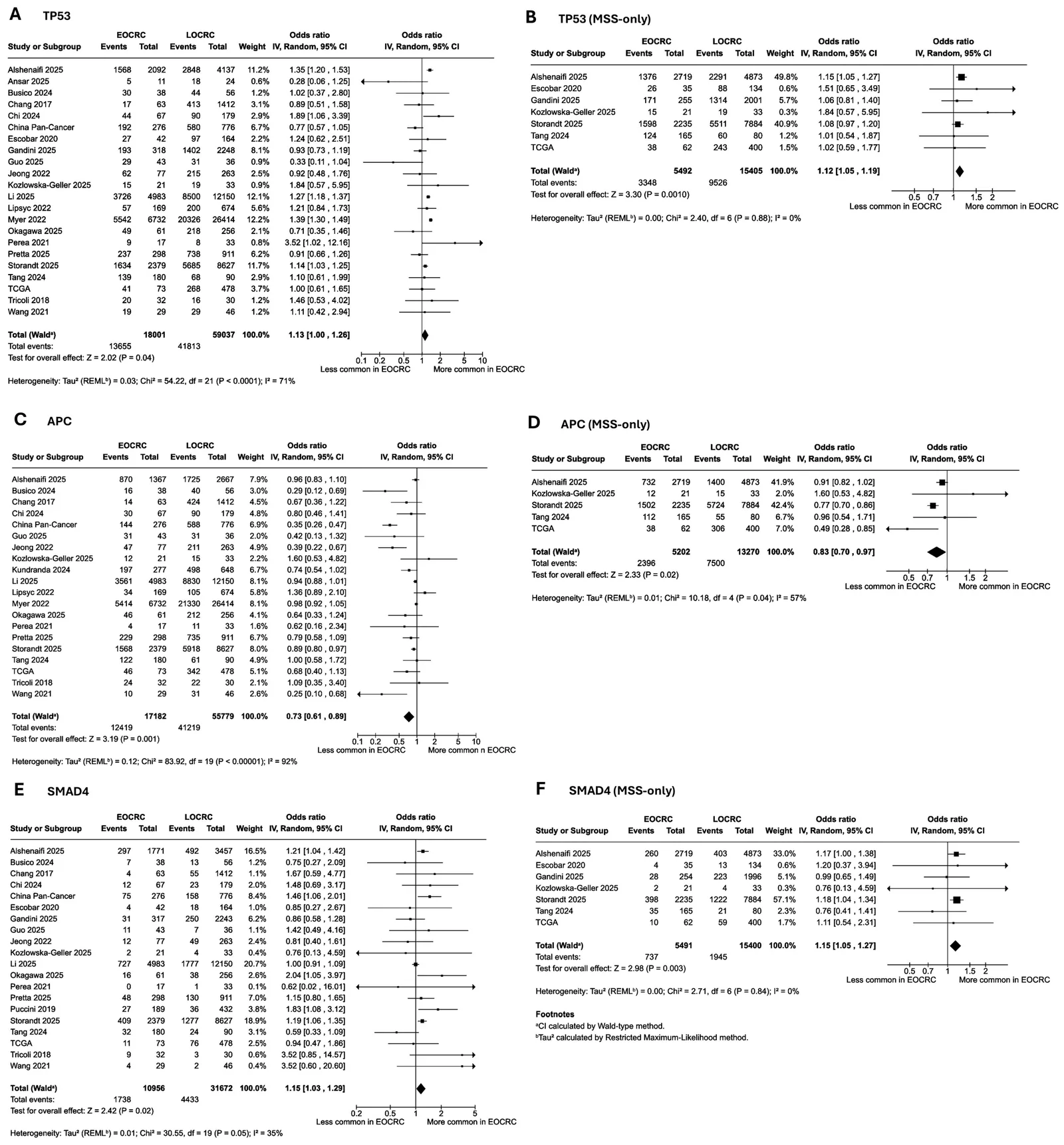
Forest plots of tumor suppressor gene mutations in EOCRC versus LOCRC. Panels (**A**,**C**,**E**) show overall analyses for *TP53*, *APC*, and *SMAD4*, respectively. Panels (**B**,**D**,**F**) show corresponding analyses restricted to microsatellite-stable (MSS) tumors. Each square represents the effect estimate for an individual study, with the area of the square proportional to the study weight; horizontal lines represent 95% confidence intervals. Diamonds denote pooled estimates with 95% confidence intervals.

Tumor suppressor genes: In the overall cohort, *TP53* mutations were more frequent (OR 1.13; 95% CI 1.00 to 1.26) and *APC* mutations less frequent (OR 0.73; 95% CI 0.61 to 0.89) in EOCRC. These differences persisted in the MSS analysis for both *TP53* (OR 1.12; 95% CI 1.05 to 1.19) and *APC* (OR 0.83; 95% CI 0.70 to 0.97) (Table 2, Figure 2). *SMAD4* mutations were also more frequent in EOCRC in both overall CRC (OR 1.15; 95% CI 1.03 to 1.29) and MSS-CRC cohorts (OR 1.15; 95% CI 1.05 to 1.27) (Table 2, Figure 2).

**Figure 3:**
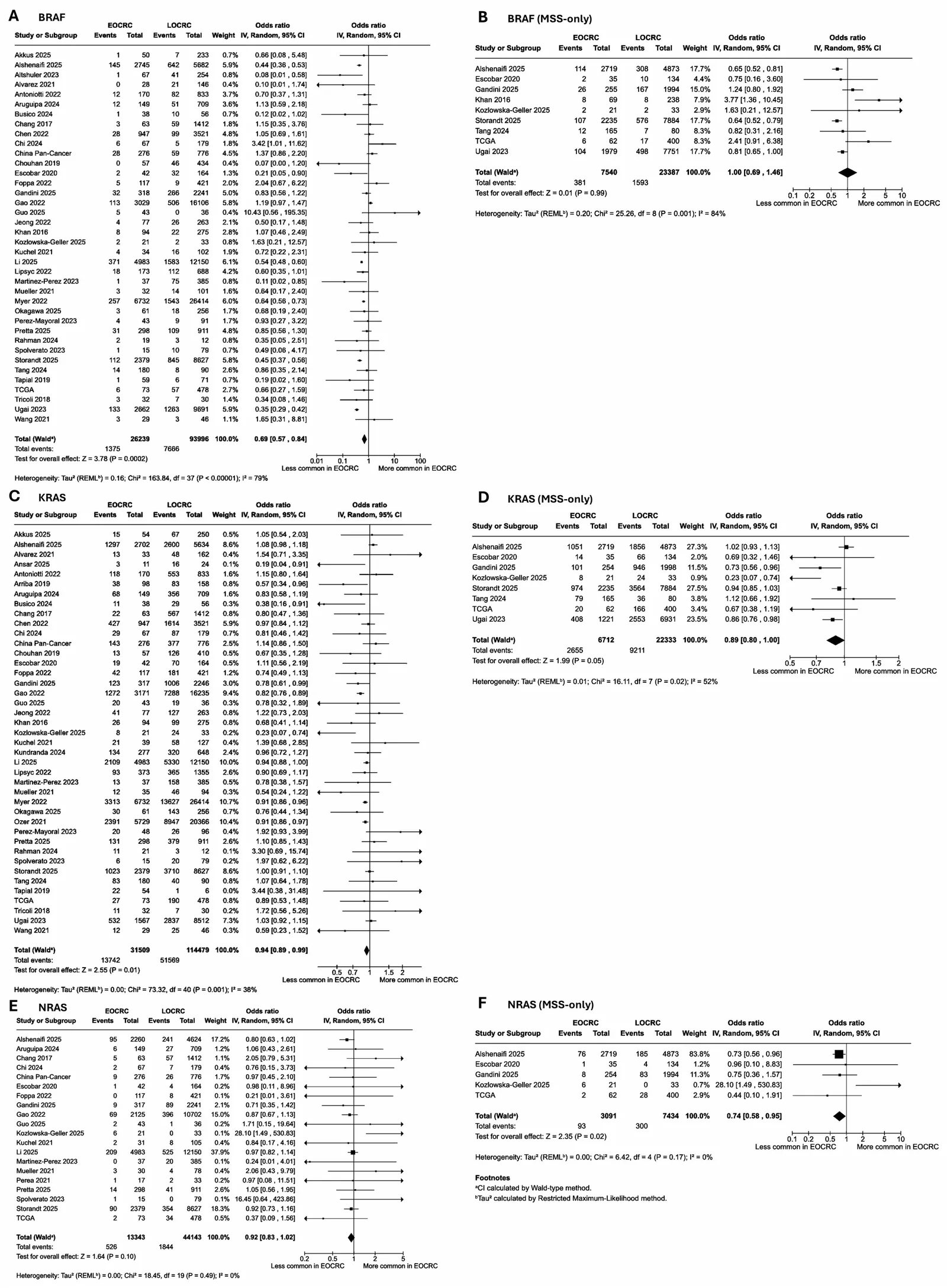
Forest plots of RAS-RAF-MAPK pathway mutations in EOCRC versus LOCRC. Panels (**A**,**C**,**E**) show overall analyses for *BRAF*, *KRAS* and *NRAS*, respectively. Panels (**B**,**D**,**F**) show corresponding analyses restricted to MSS tumors. Each square represents the effect estimate for an individual study, with the area of the square proportional to the study weight; horizontal lines represent 95% confidence intervals. Diamonds denote pooled estimates with 95% confidence intervals.

RAS-RAF-MAPK pathway: *BRAF* mutations were less frequent in EOCRC in the overall CRC cohort (OR 0.69; 95% CI 0.57 to 0.84), but this was not observed in the MSS-CRC cohort (OR 1.00; 95% CI 0.69 to 1.46) (Table 2, Figure 3). *KRAS* mutations were less frequent in EOCRC in both the overall CRC (OR 0.94; 95% CI 0.89 to 0.99) and MSS-CRC cohorts (OR 0.89; 95% CI 0.80 to 1.00) (Table 2, Figure 3). *NRAS* mutations were less frequent in EOCRC only in the MSS-CRC cohort (OR 0.74; 95% CI 0.58 to 0.95) (Table 2, Figure 3).

**Figure 4:**
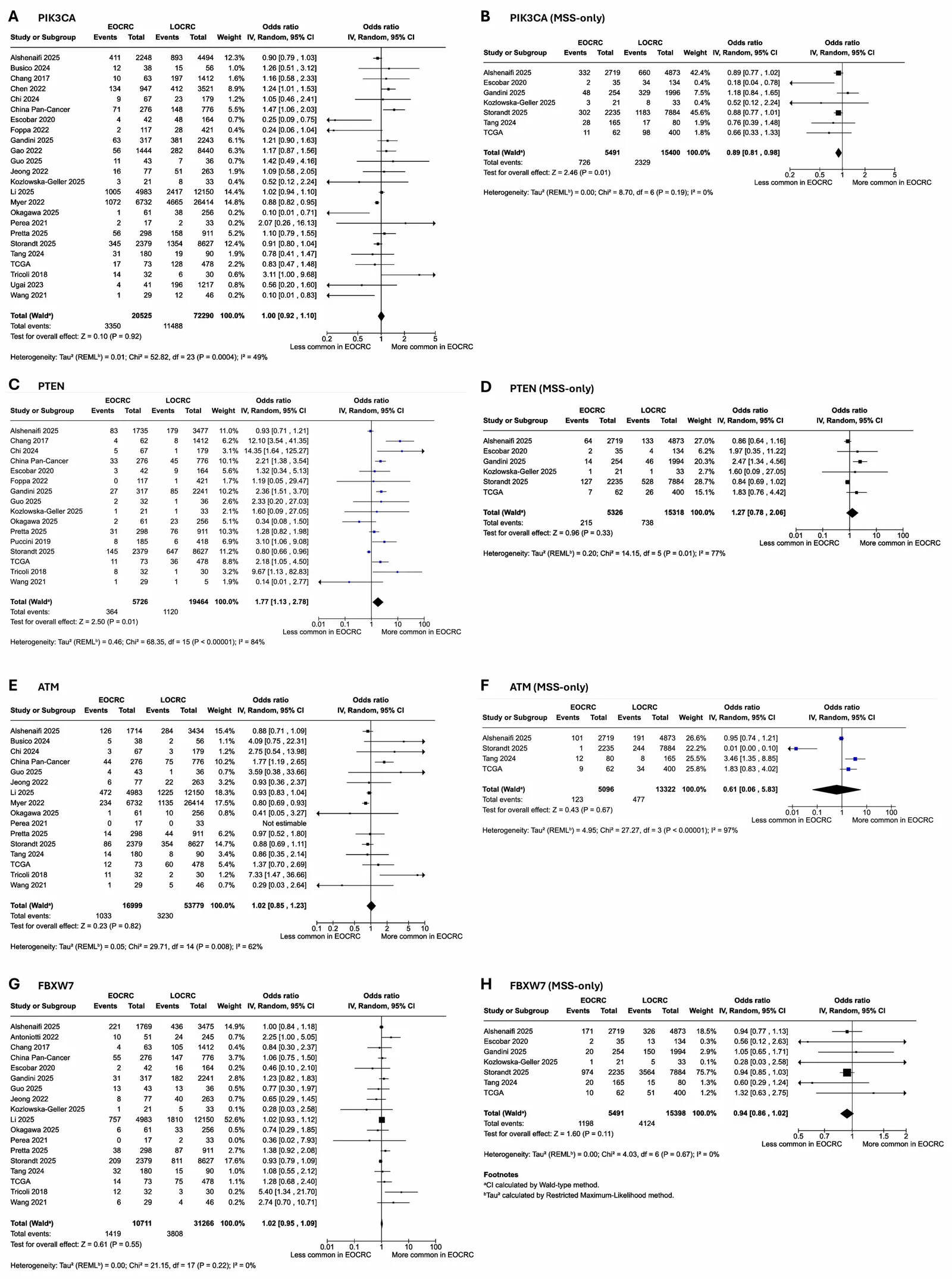
Forest plots of PI3K/AKT pathway, ATM and FBXW7 mutations in EOCRC versus LOCRC. Panels (**A**,**C**,**E**,**G**) show overall analyses for *PIK3CA*, *PTEN*, *ATM*, and FBXW7 respectively. Panels (**B**,**D**,**F**,**H**) show corresponding analyses restricted to MSS tumors. Each square represents the effect estimate for an individual study, with the area of the square proportional to the study weight; horizontal lines represent 95% confidence intervals. Diamonds denote pooled estimates with 95% confidence intervals.

PI3K/AKT pathway: *PIK3CA* mutations were less frequent in EOCRC only in the MSS-CRC cohort (OR 0.89; 95% CI 0.81 to 0.98) (Table 2, Figure 4). *PTEN* mutations were more frequent in EOCRC in the overall CRC cohort (OR 1.77; 95% CI 1.13 to 2.78), but this was not observed in the MSS-restricted analysis (OR 1.27; 95% CI 0.78 to 2.06) (Table 2, Figure 4).

There were no significant differences observed between EOCRC and LOCRC for *ATM* (OR 1.02, 95% CI 0.85 to 1.23) and *FBXW7* (OR 1.02, 95% CI 0.95 to 1.09) mutations. These findings were consistent in sensitivity and MSS-restricted analyses (Table 2, Figure 4, Supplementary Table S7).

There was significant heterogeneity for *APC*, *ATM*, *BRAF*, *PTEN*, and *TP53*. Meta-regression analyses demonstrated no significant associations between study-level differences in tumor sidedness or stage distribution and the relative mutation frequencies of *APC, ATM,* and *TP53*. For *PTEN*, tumor sidedness was not significantly associated with relative mutation frequency in univariable analysis (β = −5.55; 95% CI −11.71 to 0.61; *p* = 0.08), but a significant association was observed after adjustment for stage distribution in the multivariable model (β = −11.78; 95% CI −22.51 to −1.04; *p* = 0.03) (Table 3).

For *BRAF*, studies in which EOCRC had a relatively higher proportion of left-sided tumors compared with LOCRC demonstrated lower relative *BRAF* mutation frequency in both univariable (β = −4.67, 95% CI −7.02 to −2.32; *p* < 0.001) and multivariable analyses (β = −5.08; 95% CI −7.66 to −2.49; *p* < 0.001). Differences in stage III–IV disease distribution were not significantly associated with relative *BRAF* mutation frequency (Table 3).

Visual inspection of funnel plots suggested potential asymmetry for several gene analyses (Supplementary Figures S1–S3). Formal assessment using Egger’s regression test demonstrated evidence of funnel-plot asymmetry for *ATM* (*p* = 0.021), whereas no evidence of asymmetry was observed for *APC* (*p* = 0.124), *BRAF* (*p* = 0.541), *FBXW7* (*p* = 0.605), *KRAS* (*p* = 0.897), *NRAS* (*p* = 0.415), *PIK3CA* (*p* = 0.182), *PTEN* (*p* = 0.358), SMAD4 (*p* = 0.496), or *TP53* (*p* = 0.449). Sensitivity analyses excluding studies with total sample sizes < 100 yielded results generally similar to pooled estimates (Supplementary Table S7), supporting the robustness of the findings.

To address the potential impact of heterogeneous age cutoffs across included studies, a sensitivity analysis was performed by restricting to studies that used the definition of EOCRC as age <50 years and LOCRC as age ≥ 50 years. The results remained consistent with the primary analysis with *TP53* (OR 1.15; 95% CI 1.02 to 1.29), *APC* (OR 0.73; 95% CI 0.59 to 0.91), *SMAD4* (OR 1.15; 95% CI 1.01 to 1.31), *KRAS* (OR 0.94; 95% CI 0.90 to 0.99), *BRAF* (OR 0.71; 95% CI 0.58 to 0.88) and *PTEN* (OR 2.00; 95% CI 1.18 to 3.37) maintaining similar effect sizes and directionality (Supplementary Table S7, Supplementary Figures S4 and S5).

## 4. Discussion

This systematic review and meta-analysis represents the most updated and comprehensive synthesis to date of the mutational landscape differences between EOCRC and LOCRC. EOCRC tumors demonstrated higher frequencies of *TP53*, *SMAD4* and *PTEN* mutations, while exhibiting lower frequencies of *APC*, *KRAS* and *BRAF* mutations. When restricting analysis to MSS tumors and removing the confounding effect of hypermutation associated with MSI-H, MSS-EOCRC showed increased *TP53* and *SMAD4* mutations and decreased *APC*, *KRAS*, *NRAS* and *PIK3CA* mutations compared to LOCRC. These findings suggest that a subset of EOCRC may have a different molecular profile from LOCRC, which may have potential implications in understanding disease biology, prognostication, and therapeutic strategies.

*APC* mutations occur early in CRC carcinogenesis, and truncated APC proteins cannot effectively promote β-catenin degradation, resulting in β-catenin accumulation and activation of Wnt target genes that promote cell proliferation [66]. *TP53* maintains genomic stability, regulates cell cycle checkpoints, and induces apoptosis in response to DNA damage. *TP53* mutations may promote resistance to epidermal growth factor receptor (EGFR) inhibitors and chemotherapy drugs, which rely on p53 proteins to induce cellular apoptosis. *SMAD4* is a central mediator of the TGF-β signaling pathway and functions as a tumor suppressor by inhibiting cell proliferation and inducing apoptosis [67].

The higher frequency of *TP53* and *SMAD4* alterations and lower prevalence of *APC* mutations in younger patients may suggest that a subset of EOCRC arises through alternative pathways that are less dependent on early *APC* inactivation. Accelerated acquisition of high-impact mutations may confer aggressive growth advantages and result in more advanced stages in EOCRC.

The PI3K/AKT pathway regulates cell growth, survival and metabolism in response to growth factor signaling. *PIK3CA* mutations result in constitutive activation of PI3K and production of phosphatidylinositol-3,4,5-trisphosphate (PIP3), which activates AKT, a serine/threonine kinase that phosphorylates numerous downstream targets [66,67]. While *PIK3CA* mutations between EOCRC and LOCRC are similar in the overall CRC cohort, there was a higher frequency of *PTEN* mutations in EOCRC. *PTEN* is a negative regulator of the PI3K/AKT pathway. *PTEN* loss results in elevated PIP3 levels and constitutive AKT activation. It is associated with resistance to anti-EGFR therapy and aggressive tumor biology [66]. However, there was no difference in *PTEN* mutations when the study was limited to MSS-CRC, and this suggests that *PTEN* mutations may be associated with MSI-H tumors. The *PTEN* gene contains microsatellites within the coding region, and accumulated replication errors in MSI-H tumors may result in frameshift mutations and a non-functional *PTEN* gene [68].

The RAS-RAF-MAPK pathway is a critical signaling cascade that transduces signals from cell surface receptors to regulate cell proliferation and differentiation. *KRAS* usually occurs after *APC* inactivation [67]. The lower prevalence of RAS pathway mutations in EOCRC suggests alternative pathways that promote aggressive CRC carcinogenesis. As EOCRC patients tend to be *KRAS*-wild type, they may be suitable candidates for anti-EGFR therapy.

*BRAF* mutations result in constitutive activation of the MAPK pathway and are strongly associated with CpG island methylator phenotype (CIMP)-high status, MSI, proximal tumors, and poor prognosis [66,67]. The lower prevalence of *BRAF* mutations in EOCRC suggests that fewer young patients will experience the poor outcomes associated with *BRAF*- mutant disease. No difference in *BRAF* mutations was observed in MSS-EOCRC. This suggests that differences in the overall CRC cohort may be contributed by the MSI-H subgroup. *BRAF* mutation is associated with induction of CIMP, in which the promoters of genes are heavily methylated and silenced. This may affect the *MLH1* promoter, and loss of MLH1 protein function results in an MSI-H phenotype [67].

Our meta-regression analysis also demonstrated an association between study-level differences in tumor anatomical distribution and the relative prevalence of *BRAF* mutations between EOCRC and LOCRC. Studies in which EOCRC had a relatively higher proportion of left-sided tumors demonstrated lower relative *BRAF* mutation prevalence, and this association remained significant after adjustment for study-level differences in stage distribution. In our pooled cohort, EOCRC patients had significantly more left-sided and rectal tumors, which are less likely to harbor *BRAF* mutations regardless of age. The lower *BRAF* frequency in EOCRC may reflect anatomical distribution differences rather than age-specific biology alone. A significant association between study-level differences in tumor sidedness and relative *PTEN* mutation frequency was also observed in the multivariable meta-regression after adjustment for stage distribution; however, this association was not significant in the univariable analysis. Given the substantial residual heterogeneity and the study-level nature of the analysis, this finding should be considered exploratory and requires confirmation using individual patient-level data.

The anatomical distribution difference is particularly important, as left-sided and rectal cancers have different molecular profiles, treatment approaches, and prognoses compared to right-sided tumors. The higher proportion of left-sided disease in EOCRC may also explain the mutational profile observed. Left-sided tumors are more likely to be chromosomally unstable, *TP53*-mutant, *KRAS*-wildtype and responsive to anti-EGFR therapy, while right-sided tumors are more likely to be MSI-H, *BRAF*-mutant, and have distinct immune microenvironments [67].

Several alternative mechanisms have been proposed for colorectal carcinogenesis in young adults. The microbiome dysbiosis theory proposes that alterations in the gut microbiome promote chronic inflammation, produce genotoxic metabolites and disrupt intestinal barrier function [69]. It is postulated that changes in dietary patterns such as high consumption of red and processed meats or early antibiotic exposure may promote microbial dysbiosis. *Escherichia coli* strains that are *pks+* produce colibactin, a genotoxin that induces DNA damage. Previous mutational signature analysis demonstrated that the SBS88 signature, associated with colibactin-induced DNA damage, is more common in EOCRC [70].

Another proposed mechanism would be metabolic dysfunction, given the rising incidence of obesity and metabolic syndrome in parallel with the rising incidence of EOCRC. Obesity is associated with chronic inflammation, insulin resistance and altered adipokine secretion. Elevated levels of insulin-like growth factor 1 (IGF-1) in obesity can promote cellular proliferation and metabolic dysregulation in cancer [71]. Leptin levels are elevated in obesity and can activate JAK/STAT, MAPK and PI3K/AKT pathways that promote proliferation and angiogenesis [72]. Obesity is also characterized by chronic low-grade systemic inflammation with elevated levels of pro-inflammatory cytokines such as TNF-α, IL-6 and IL-1β. This pro-inflammatory environment may induce oxidative stress, activate proliferative signaling pathways and suppress anti-tumor immune response [73]. Insulin-IGF-1 signaling activates MAPK and PI3K/AKT pathways independent of *KRAS* mutation. This is a potential mechanism in which EOCRC tumors maintain downstream activation of proliferative pathways despite lower *KRAS* mutational frequency. Lower *APC* mutational prevalence in EOCRC may reflect alternative Wnt pathway activation through inflammatory mediators produced by microbial dysbiosis and metabolic dysfunction rather than canonical *APC* loss.

## 5. Clinical Implications

There are several clinical implications based on this study’s findings. The higher prevalence of *TP53* and *SMAD4* mutations in MSS-EOCRC can be considered as poor prognostic markers, and tumors may exhibit potential resistance to 5-fluorouracil-based chemotherapy. The lower prevalence of *KRAS* and *NRAS* mutations, especially in MSS-EOCRCs, suggests that a modestly higher proportion of EOCRC patients may be potential candidates for anti-EGFR therapy. However, the use of anti-EGFR therapy is dependent on multiple factors apart from *KRAS* mutation status, such as tumor sidedness, tumor stage, *BRAF* mutation status and MSI status. Higher prevalence of MSI-H in EOCRC suggests that more young patients may benefit from immune checkpoint inhibitors. This finding supports the need for universal MMR testing in all newly diagnosed CRC patients, especially for young patients, to identify suitable candidates for immunotherapy.

## 6. Future Directions

The lower prevalence of *APC* and *KRAS* and higher prevalence of *TP53* and *SMAD4* mutations in EOCRC suggests that age-related differences in mutational profiles may exist. However, these differences were generally modest in magnitude, and there was substantial between-study heterogeneity observed for several genes. There is a need for prospective, standardized genomic studies to validate this study’s findings. Additionally, future research using comprehensive genomic (e.g., whole-exome or whole-genome sequencing), epigenomic, and transcriptomic profiling of large EOCRC cohorts can identify novel drivers and pathways. Integration with tumor microenvironment and gut microbiome analysis may provide additional insights into microbial–immune interactions. Certain microbes may promote carcinogenesis through chronic inflammation or modulation of the host immune response [74]. Functional validation in patient-derived models will be essential to translate these findings into therapeutic strategies.

## 7. Strengths

This study has several notable strengths that distinguish it from previous studies. Firstly, the study represents a comprehensive synthesis of EOCRC mutational profiles, with 154,982 patients from 43 primary studies and 2 major public databases. However, while the large sample size in this meta-analysis provides substantial statistical power to detect differences between EOCRC and LOCRC, these significant molecular differences are associated with relatively modest effect sizes, and clinical implications of these findings must be carefully considered. Secondly, MSS-specific subgroup analysis addressed the potential confounding effect of microsatellite status in comparative genomic studies between EOCRC and LOCRC. By isolating mutational differences attributable to age-related biology rather than microsatellite status, our subgroup analysis reflects true age-specific observations. Thirdly, meta-regression was used to evaluate study-level differences in tumor sidedness and stage distribution as potential sources of heterogeneity, identifying an association between greater relative left-sided tumor predominance in EOCRC and lower relative *BRAF* mutation frequency. Fourthly, studies with overlapping cohorts or analyzed public databases were excluded to ensure independence of observations. The approach to include mutational data from TCGA and China PanCancer from cBioPortal ensures that landmark datasets were included in the final analysis while removing duplicate sampling, which may affect the precision of the analysis. Fifth, our gene panel included 10 key driver genes spanning multiple critical pathways in colorectal carcinogenesis, such as the RAS-RAF-MAPK and PI3K/AKT pathways. This ensured systematic and standardized coverage of therapeutically relevant alterations. Finally, sensitivity analysis excluding studies with sample sizes under 100 confirmed the robustness of the study findings and mitigated concerns regarding small-study effects.

## 8. Limitations

Several limitations should be acknowledged. Substantial residual heterogeneity observed for *APC*, *ATM*, *PTEN* and *TP53* despite meta-regression may be due to varying age definitions, different sequencing methods, patient populations and ethnic diversity. Differences in sequencing platform, depth, coverage, variant call pipelines and histological subtypes may contribute to inter-study variability. Most studies also did not report on the exclusion of hereditary CRC syndromes, which are associated with distinct mutational profiles and may inflate the prevalence of certain mutations in EOCRC. These factors limit the precision of pooled estimates and underscore the need for standardized, prospective genomic analyses. Additionally, the included studies utilized both formalin-fixed paraffin-embedded (FFPE) and fresh-frozen tissue samples, which differ in DNA quality and may affect the detection of variants. These limit the precision of our estimates and may obscure true biological differences. Our analysis was restricted to 10 common driver genes, which may not identify novel mutations that can contribute to earlier carcinogenesis. The analysis was also limited to mutational frequency without assessing the functional significance of individual mutations. The included studies did not consistently report whether mutations were oncogenic drivers or passenger mutations, and this may obscure important differences in the biological significance of mutations between EOCRC and LOCRC. Future studies with functional annotation of variants are needed to determine whether observed differences reflect driver events or differences in passenger mutation burden.

Although Egger’s regression test showed no evidence of funnel-plot asymmetry for most gene analyses, evidence of small-study effects was observed for ATM. Funnel-plot asymmetry may arise from publication bias, between-study heterogeneity, or other study-level factors and should therefore not be interpreted as definitive evidence of publication bias. Furthermore, although sensitivity analyses excluding smaller studies yielded largely consistent pooled estimates, these analyses cannot exclude the possibility of publication bias. Furthermore, meta-regression was based on study-level rather than individual patient-level data. Accordingly, observed associations between clinicopathological characteristics and mutational differences are susceptible to ecological bias and should not be interpreted as causal. Finally, the majority of the included studies utilized targeted sequencing panels, which often detect mutations in samples from patients diagnosed with locally advanced or metastatic CRC. This may introduce a selection bias and may not fully represent the mutational landscape of EOCRC tumors.

## 9. Conclusions

This comprehensive systematic review and meta-analysis identified differences in mutational prevalence of known somatic CRC driver genes between EOCRC and LOCRC even after adjustment for microsatellite status. These molecular profiles, combined with significant clinicopathological differences, suggest that a subset of EOCRC may involve alternative carcinogenesis pathways rather than simply an earlier manifestation of the same disease process. However, this should be interpreted with caution given the modest effect sizes and substantial heterogeneity between studies. These findings are interpreted as hypothesis-generating rather than definitive evidence that EOCRC represents a biologically distinct disease entity. As the incidence of EOCRC continues to rise globally, understanding its unique biology becomes increasingly urgent. Future research should focus on understanding the biological basis of these molecular differences and translate these molecular insights into improved prevention, early detection, and treatment strategies for young patients with colorectal cancer.

## Figures and Tables

**Table 1:** Clinicopathological characteristics of the pooled studies.

	No. of Studies Included	EOCRC, No. (%)	LOCRC, No. (%)
TNM Stage	28	*n* = 12,050	*n* = 53,050
I/II		3529 (29.3)	20,293 (38.3)
III/IV		8521 (70.7)	32,757 (61.7)
Tumor location	31	*n* = 15,103	*n* = 47,031
Right		5844 (38.7)	20,508 (43.6)
Left/Rectum		9259 (61.3)	26,523 (56.4)
Grade of differentiation	16	*n* = 8,066	*n* = 39,327
Well-moderate		6371 (79.0)	33,823 (86.0)
Poor		1695 (21.0)	5504 (14.0)
MSI Status	35	*n* = 15,765	*n* = 67,009
MSI		2052 (13.1)	6020 (9.0)
MSS		13,713 (87.0)	60,989 (91.0)

Footnote: Values represent aggregated counts and proportions from studies reporting each clinicopathological characteristic. These data are presented descriptively and were not subjected to inferential statistical testing, as aggregation across studies does not account for between-study heterogeneity.

**Table 2:** Summary of meta-analysis results that compare the prevalence of somatic mutations in EOCRC with LOCRC.

Classification	Gene	All Studies				Studies that Analyzed Only MSS-CRC			
		No. of Studies	OR (95% CI)	*p*-Value	I^2^ (%)	No. of Studies	OR (95% CI)	*p*-Value	I^2^ (%)
Tumor Suppressors	*TP53*	22	1.13 (1.00–1.26)	0.04	71	7	1.12 (1.05–1.19)	0.001	0
	*APC*	20	0.73 (0.61–0.89)	0.001	92	5	0.83 (0.70–0.97)	0.02	57
	*SMAD4*	20	1.15 (1.03–1.29)	0.02	35	6	1.15 (1.05–1.27)	0.003	0
	*ATM*	16	1.02 (0.85–1.23)	0.82	62	4	0.61 (0.06–5.83)	0.67	97
	*FBXW7*	18	1.02 (0.95–1.09)	0.55	0	7	0.94 (0.86–1.02)	0.11	0
RAS-RAF-MAPK	*BRAF*	38	0.69 (0.57–0.84)	0.0002	79	9	1.00 (0.69–1.46)	0.99	84
	*KRAS*	41	0.94 (0.89–0.99)	0.01	38	8	0.89 (0.80–1.00)	0.05	52
	*NRAS*	20	0.92 (0.83–1.02)	0.10	0	5	0.74 (0.58–0.95)	0.02	0
PI3K/AKT	*PIK3CA*	24	1.00 (0.92–1.10)	0.92	49	7	0.89 (0.81–0.98)	0.01	0
	*PTEN*	16	1.77 (1.13–2.78)	0.01	84	6	1.27 (0.78–2.06)	0.33	77

**Table 3:** Meta-regression analysis by adjusting for tumor sidedness and stage distribution.

Gene	Moderator	Univariate				Multivariate				
		Beta-Coefficient, 95%CI	*p*-Value	R^2^ (%)	Residual I^2^ (%)	Beta-Coefficient, 95%CI	*p*-Value	Overall *p*-Value	R^2^ (%)	Residual I^2^ (%)
APC	Left-sided tumor Stage III/IV	−1.66 (−4.76, 1.45) −0.58 (−4.31, 3.14)	0.30 0.76	0.00 0.00	78.80 89.76	−2.89 (−8.33, 2.56) 0.66 (−4.07, 5.40)	0.30 0.78	0.56	0.00	81.70
ATM	Left-sided tumor Stage III/IV	−0.71 (−2.98, 1.55) −0.58 (−4.49, 3.33)	0.54 0.77	0.00 0.00	0.00 66.48	−1.64 (−10.19, 6.90) 1.11 (−7.58, 5.35)	0.71 0.74	0.88	0.00	74.85
BRAF	Left-sided tumor Stage III/IV	−4.67 (−7.02, −2.32) −0.85 (−4.28, 2.58)	<0.001 0.63	63.65 0.00	45.63 83.61	−5.08 (−7.66, −2.49) 0.82 (−2.20, 3.83)	<0.001 0.60	<0.001	77.95	28.35
KRAS	Left-sided tumor Stage III/IV	−0.03 (−0.89, 0.82) −1.23 (−2.51, 0.05)	0.94 0.059	0.00 25.09	17.95 35.78	0.58 (−0.27, 1.42) −0.60 (−1.75, 0.55)	0.18 0.30	0.12	99.97	0.00
PTEN	Left-sided tumor Stage III/IV	−5.55 (−11.71, 0.61) −0.48 (−7.24, 6.28)	0.08 0.89	16.16 0.00	88.82 88.42	−11.78 (−22.51,−1.04) 7.48 (−1.38,16.35)	0.03 0.10	0.10	43.92	87.60
TP53	Left-sided tumor Stage III/IV	−0.37 (−1.37, 2.11) 0.93 (−0.85, 2.72)	0.68 0.31	0.00 2.19	48.22 51.97	0.42 (−1.88, 2.73) 0.13 (−1.95, 2.22)	0.72 0.90	0.91	0.00	37.87

## Data Availability

All data supporting the findings of this study are available within the article and its supplementary information files.

## Supplementary Materials

The following supporting information can be downloaded at: https://www.mdpi.com/article/10.3390/cancers18193225/s1, Figure S1: Assessment of publication bias and small-study effects for tumor suppressor gene mutations. Figure S2: Assessment of publication bias and small-study effects for RAS-RAF-MAPK pathway mutations. Figure S3: Assessment of publication bias and small-study effects for PI3K/AKT pathway, ATM and FBXW7 mutations. Figure S4: Sensitivity analysis for genes TP53, APC, SMAD4, BRAF, KRAS, NRAS in which studies that defined EOCRC as age ≤ 50 years were excluded. Figure S5: Sensitivity analysis for genes PIK3CA, PTEN, ATM and FBXW7 in which studies that defined EOCRC as age ≤ 50 years were excluded. Table S1: Search strategy for PubMed & Embase. Table S2: List of articles excluded as the study cohort overlapped with public cohorts and studies. Table S3: Characteristics of included studies and public datasets. Table S4: Assessment of quality of cohort studies with Joanna Briggs Institute Tools. Table S5: Assessment of quality of randomized controlled trials with Joanna Briggs Institute Tools. Table S6: Assessment of quality of cross-sectional studies with Joanna Briggs Institute Tools. Table S7: Sensitivity analyses in which small studies with sample size < 100 were excluded and studies that defined EOCRC as age ≤ 50 years were excluded.
