# Supplementary material for: Differences in Somatic Mutations Between Early-Onset and Late-Onset Colorectal Cancer: A Systematic Review and Meta-Analysis
Source: Cancers (Basel). 2026 Oct 7;18(19):3225. doi: 10.3390/cancers18193225 (PMC13664542; doi:10.3390/cancers18193225)
Supplement: Supplementary file 1 [file cancers-18-03225-s001.zip › cancers-4551013-supplementary.pdf]

Supplementary Table S1: Search strategy for PubMed & Embase.

Search #	Concept	Search Terms (PubMed)	Search Terms (Embase)
1	CRC	(“colorectal neoplasms”[mesh] OR “colorectal ca ncer” OR “colonic neoplasms”[mesh] OR “colon c ancer” OR “rectal neoplasms”[mesh] or “rectal ca ncer”)	(‘colon tumor’/exp OR ‘rectum tumor’/exp OR ‘colorectal tumor’/exp OR colorectal:ti,ab OR colon:ti,ab OR rectal:ti,ab) AND (cancer:ti,ab OR carcinoma:ti,ab OR adenocarcinoma:ti,ab OR tumor*:ti,ab OR tumour*:ti,ab OR neoplas*:ti,ab)
2	Early-onset	(“age of onset”[mesh] OR “early-onset”[tiab] OR “early onset”[tiab] OR “young-onset”[tiab] OR “y oung onset”[tiab] OR “50 years”[tiab] or “age 50”[tiab])	((‘age of onset’/exp OR ‘young adult’/exp) OR ((early AND onset:ti,ab OR ‘early onset’:ti,ab OR young) AND onset:ti,ab) OR ‘young onset’:ti,ab OR eocrc:ti,ab OR ‘yo crc’:ti,ab OR (young AND adult*:ti,ab) OR adolescent:ti,ab OR ((age:ti,ab OR aged:ti,ab OR ages:ti,ab) AND (young:ti,ab OR ‘under 50’:ti,ab OR ‘(50’:ti,ab OR ‘less than 50’:ti,ab)))
3	Mutational Profile	(“Mutation”[Mesh] OR “mutational profile”[tiab] OR “mutational landscape”[tiab] OR “genomic profile”[tiab] OR “genetic profile”[tiab] OR “molecular profile”[tiab] OR “somatic mutation”[tiab] OR “driver mutation”[tiab] OR “gene mutation”[tiab] OR “next-generation sequencing”[tiab] OR “NGS”[tiab] OR “targeted sequencing”[tiab] OR “targeted next-generation sequencing”[tiab] OR “targeted NGS”[tiab] OR “gene panel”[tiab] OR “custom panel”[tiab] OR “whole exome”[tiab] OR “whole genome”[tiab] OR “MSI”[tiab] OR “microsatellite instability”[tiab])	(‘mutation’/exp OR ‘mutational profile’:ti,ab OR ‘mutational landscape’:ti,ab OR ‘genomic profile’:ti,ab OR ‘genetic profile’:ti,ab OR ‘molecular profile’:ti,ab OR ‘somatic mutation’:ti,ab OR ‘driver mutation’:ti,ab OR ‘gene mutation’:ti,ab OR ‘next-generation sequencing’/exp OR ‘next-generation sequencing’:ti,ab OR ‘ngs’:ti,ab OR ‘targeted sequencing’:ti,ab OR ‘targeted next-generation sequencing’:ti,ab OR ‘targeted ngs’:ti,ab OR ‘gene panel’:ti,ab OR ‘custom panel’:ti,ab OR ‘whole exome sequencing’/exp OR ‘whole exome’:ti,ab OR ‘whole genome sequencing’/exp OR ‘whole genome’:ti,ab OR ‘msi’:ti,ab OR ‘microsatellite instability’/exp OR ‘microsatellite instability’:ti,ab)
Combined	#1 AND #2 AND #3		

Supplementary Table S2: List of articles excluded as the study cohort overlapped with public cohorts and studies

**Article**	**Author**	**Publication Year**	**Overlapping cohort and studies**
Clinical significance of frequent somatic mutations detected by high-throughput targeted sequencing in archived colorectal cancer samples	Dallol A, et al.	2016	GENIE
Early-onset Colorectal Cancer is Distinct From Traditional Colorectal Cancer.	Yeo H, et al.	2017	TCGA
Associations between Loss of ARID1A Expression and Clinicopathologic and Genetic Variables in T1 Early Colorectal Cancer	Kishida Y, et al.	2019	GENIE
Clinical and molecular comparative study of colorectal cancer based on age-of-onset and tumor location: Two main criteria for subclassifying colorectal cancer	Álvaro E, et al.	2019	Perea 2021
Clinicopathological and mutational analyses of colorectal cancer with mutations in the POLE gene	Hino H, et al.	2019	GENIE
Association between risk factors, molecular features and CpG island methylator phenotype colorectal cancer among different age groups in a Taiwanese cohort	Chen KH, et al.	2021	GENIE
The prognostic impact of RAS on overall survival following liver resection in early versus late-onset colorectal cancer patients	Jácome AA, et al.	2021	Aruguipa 2024
A Comprehensive Comparison of Early-Onset and Average-Onset Colorectal Cancers	Cercek A, et al.	2021	Li 2025
Tumor long interspersed nucleotide element-1 (LINE-1) hypomethylation in relation to age of colorectal cancer diagnosis and prognosis	Akimoto N, et al.	2021	Ugai 2022
Racial/Ethnic and Sex Differences in Somatic Cancer Gene Mutations among Patients with Early-Onset Colorectal Cancer	Holowatyj AN, et al.	2023	GENIE
Investigating the WNT and TGF-beta pathways alterations and tumor mutant burden in young-onset colorectal cancer	Ferrell M, et al.	2024	GENIE
Artificial Intelligence-Guided Molecular Determinants of PI3K Pathway Alterations in Early-Onset Colorectal Cancer Among High-Risk Groups Receiving FOLFOX	Diaz FC, et al.	2025	TCGA, GENIE

Comprehensive assessment of genomic heterogeneity, coalterations, and outcomes of patients with colorectal cancer: An AACR GENIE project analysis	Stecko H, et al.	2025	GENIE
Detecting PI3K and TP53 Pathway Disruptions in Early-Onset Colorectal Cancer Among Hispanic/Latino Patients	Monge C, et al.	2025	GENIE
Ethnicity-Specific Molecular Alterations in MAPK and JAK/STAT Pathways in Early-Onset Colorectal Cancer	Monge C, et al.	2025	GENIE
Integration of multi-omics data to unveil the molecular landscape and role of piRNAs in early-onset colorectal cancer	Zhou S, et al.	2025	TCGA
Molecular Heterogeneity in Early-Onset Colorectal Cancer: Pathway-Specific Insights in High-Risk Populations	Monge C, et al.	2025	GENIE
Precision Oncology Insights into WNT Pathway Alterations in FOLFOX-Treated Early-Onset Colorectal Cancer in High-Risk Populations	Diaz FC, et al.	2025	TCGA, GENE
Early onset metastatic colorectal cancer patients as a distinctive clinical and molecular phenomenon.	Pretta A, et al.	2025	Pretta 2025

Supplementary Table S3*: *Characteristics of included studies and public datasets (NR: Not Reported).

Author	Publication Year	Country	Sequencing Method	Age Definition (EOCRC)	Age Definition (LOCRC)	Cohort sequenced (n)	EOCRC sequenced (n)	LOCRC sequenced (n)	Exclusion of Hereditary CRC or IBD
Khan [24]	2016	USA	Targeted	£30	^{3}50	369	94	275	NR
Chang [25]	2017	Taiwan	Targeted	<50	^{3}50	1475	63	1412	Hereditary CRC excluded
Tricoli [26]	2018	USA	WES	<40	>60	62	32	30	NR
Arriba [27]	2019	Spain	Targeted	£50	^{3}70	272	98	174	Hereditary CRC & IBD excluded
Chouhan [28]	2019	USA	Targeted	£50	>50	497	57	440	Hereditary CRC & IBD excluded
Puccini [29]	2019	USA	Targeted	<45	^{3}65	1126	350	776	NR
Tapial [30]	2019	Spain	Targeted	£45	>70	130	59	71	NR
Escobar [31]	2020	USA	Targeted	<40	>70	206	42	164	NR
Alvarez [32]	2021	Chile	Targeted	£50	>50	426	72	354	NR
Kuchel [33]	2021	Australia	Targeted	£50	>50	187	42	145	NR
Mueller [34]	2021	Switzerland	Targeted	<50	^{3}50	411	70	341	Not excluded
Ozer [35]	2021	USA	Targeted	<50	^{3}50	26095	5729	20366	NR
Perea [36]	2021	Spain	Targeted	<50	^{3}69	50	17	33	Hereditary CRC & IBD excluded
Wang [37]	2021	China	Targeted	£50	>50	75	29	46	NR
Antoniotti [38]	2022	Italy	Targeted	<50	^{3}50	296	51	245	NR
Chen [39]	2022	China	Targeted	<50	^{3}50	4468	947	3521	NR

Foppa [40]	2022	Italy	Targeted	<50	^{3}50	538	117	421	Hereditary CRC & IBD excluded
Gao [41]	2022	China	Targeted	<50	^{3}50	19410	3171	16239	NR
Jeong [42]	2022	Korea	Targeted	<50	^{3}50	340	77	263	NR
Lipsyc-Sharf [43]	2022	Multicentre (USA, Canada)	Targeted	<50	^{3}50	2326	514	1812	NR
Myer [44]	2022	USA	Targeted	<50	^{3}50	33146	6732	26414	NR
Ugai [45]	2022	USA	Targeted	<50	^{3}55	1552	35	1517	NR
Altshuler [46]	2023	USA	Targeted	£50	>50	321	67	254	NR
Martinez-Perez [47]	2023	Spain	Targeted	£50	>50	422	37	385	NR
Perez-Mayoral [48]	2023	Puerto Rico	Targeted	<50	^{3}50	192	72	120	Hereditary CRC & IBD excluded
Spolverato [49]	2023	Italy	Targeted	£50	>50	94	15	79	IBD excluded
Ugai [50]	2023	Multicentre (USA, Germany, Australia, Sweden, Canada)	Targeted	<50	^{3}50	14004	3089	10915	NR
Aruquipa [51]	2024	Brazil	Targeted	<50	^{3}50	858	149	709	NR
Busico [52]	2024	Italy	Targeted	<40	>60	94	38	56	Hereditary CRC excluded
Chi [53]	2024	China	Targeted	<50	^{3}50	246	67	179	NR

Kundranda [54]	2024	USA	WES	£50	>50	925	277	648	NR
Rahman [55]	2024	USA	Targeted	<50	>65	88	50	38	NR
Tang [56]	2024	China	Targeted	<50	³50	270	180	90	NR
Akkus [57]	2025	Turkey	Targeted	<50	³50	568	90	478	NR
Alshenaifi [58]	2025	USA	Targeted	<50	³50	9032	2895	6137	NR
Ansar [59]	2025	Pakistan	Targeted	£50	>50	35	11	24	NR
Gandini [60]	2025	France	Targeted	<50	50	2973	366	2607	NR
Guo [61]	2025	China	WES	<50	³50	81	45	36	NR
Kozlowska-Geller [62]	2025	Poland	Targeted	£50	>50	54	21	33	NR
Li [14]	2025	Multicentre (Canada, China, France, Nigeria, South Korea, Spain, Netherlands, USA (8 countries))	WES	<50	³50	17133	4983	12150	Hereditary CRC excluded
Okagawa [63]	2025	Japan	Targeted	<50	³50	317	61	256	Hereditary CRC & IBD excluded
Pretta [64]	2025	Italy	Targeted	£50	>50	1209	298	911	NR
Storandt [65]	2025	USA	Targeted	<50	³50	11006	2379	8627	NR
China PanCancer Atlas [21]		China	Targeted	<50	≥50	1052	276	776	NR

TCGA [20]		USA	WES/WGS	<50	³50	551	73	478	NR

Supplementary Table S4: Assessment of quality of cohort studies with Joanna Briggs Institute Tools.

Author	Publication Year	Q1	Q2	Q3	Q4	Q5	Q6	Q7	Q8	Q9	Q10	Q11	Risk of Bias
Khan	2016	Yes	Yes	Yes	Yes	Yes	Yes	Yes	Yes	No	No	Yes	Low
Chang	2017	Yes	Yes	Yes	Yes	Yes	Yes	Yes	Yes	No	No	Yes	Low
Arriba	2019	Yes	Yes	Yes	Yes	No	Yes	Yes	Yes	No	No	Yes	Moderate
Chouhan	2019	Yes	Yes	Yes	Yes	No	Yes	Yes	No	No	No	Yes	Moderate
Escobar	2020	Yes	Yes	Yes	Yes	No	Yes	Yes	No	No	No	Yes	Moderate
Alvarez	2021	Yes	Yes	Yes	Yes	No	Yes	Yes	Yes	No	No	Yes	Moderate
Kuchel	2021	Yes	Yes	Yes	Yes	Yes	Yes	Yes	Yes	No	No	Yes	Low
Mueller	2021	Yes	Yes	Yes	Yes	No	Yes	Yes	Yes	No	No	Yes	Moderate
Ozer	2021	Yes	Yes	Yes	Yes	Yes	Yes	Yes	Yes	No	No	Yes	Low
Perea	2021	Yes	Yes	Yes	Yes	No	Yes	Yes	Yes	No	No	Yes	Moderate
Wang	2021	Yes	Yes	Yes	Yes	No	Yes	Yes	No	No	No	Yes	Moderate
Chen	2022	Yes	Yes	Yes	Yes	No	Yes	Yes	No	No	No	Yes	Moderate
Foppa	2022	Yes	Yes	Yes	Yes	Yes	Yes	Yes	Yes	No	No	Yes	Low
Jeong	2022	Yes	Yes	Yes	Yes	No	Yes	Yes	Yes	No	No	Yes	Moderate
Martinez-Perez	2023	Yes	Yes	Yes	Yes	Yes	Yes	Yes	Yes	No	No	Yes	Low
Spolverato	2023	Yes	Yes	Yes	Yes	No	Yes	Yes	Yes	No	No	Yes	Moderate
Busico	2024	Yes	Yes	Yes	Yes	No	Yes	Yes	No	No	No	Yes	Moderate
Rahman	2024	Yes	Yes	Yes	Yes	Yes	Yes	Yes	Yes	No	No	Yes	Low
Tang	2024	Yes	Yes	Yes	Yes	No	Yes	Yes	Yes	No	No	Yes	Moderate
Akkus	2025	Yes	Yes	Yes	Yes	Yes	Yes	Yes	Yes	No	No	Yes	Low
Alshenaifi	2025	Yes	Yes	Yes	Yes	Yes	Yes	Yes	No	No	No	Yes	Moderate
Guo	2025	Yes	Yes	Yes	Yes	Yes	Yes	Yes	Yes	No	No	Yes	Low

Kozlowska-Geller	2025	Yes	Yes	Yes	Yes	Yes	Yes	Yes	No	No	No	Yes	Moderate
Pretta	2025	Yes	Yes	Yes	Yes	Yes	Yes	Yes	Yes	No	No	Yes	Low

JBI Critical Appraisal Checklist for Cohort Studies:

1) Were the two groups similar and recruited from the same population?
2) Were the exposures measured similarly to assign people to both exposed and unexposed groups?
3) Was the exposure measured in a valid and reliable way?
4) Were confounding factors identified?
5) Were strategies to deal with confounding factors stated?
6) Were the groups/participants free of the outcome at the start of the study (or at the moment of exposure)?
7) Were the outcomes measured in a valid and reliable way?
8) Was the follow up time reported and sufficient to be long enough for outcomes to occur?
9) Was follow up complete, and if not, were the reasons to loss to follow up described and explored?
10) Were strategies to address incomplete follow up utilized?
11) Was appropriate statistical analysis used?

Supplementary Table S5: Assessment of quality of randomized controlled trials with Joanna Briggs Institute Tools.

Author	Publication Year	Q1	Q2	Q3	Q4	Q5	Q6	Q7	Q8	Q9	Q10	Q11	Q12	Q13	Risk of Bias
Antoniotti	2022	Yes	Yes	Yes	No	No	Yes	No	Yes	Yes	Yes	Yes	Yes	Yes	Low
Lipsyc-Sharf	2022	Yes	No	Yes	No	No	Yes	No	Yes	Yes	Yes	Yes	Yes	Yes	Moderate
Gandini	2025	Yes	No	Yes	No	No	Yes	No	Yes	Yes	Yes	Yes	Yes	Yes	Moderate

JBI Critical Appraisal Checklist for RCT

1. Was true randomization used for assignment of participants to treatment groups?
2. Was allocation to treatment groups concealed?
3. Were treatment groups similar at the baseline?
4. Were participants blind to treatment assignment?
5. Were those delivering treatment blind to treatment assignment?
6. Were treatment groups treated identically other than the intervention of interest?
7. Were outcomes assessors blind to treatment assignment?
8. Were outcomes measured in the same way for treatment groups?
9. Were outcomes measured in a reliable way?
10. Was follow up complete and if not, were differences between groups in terms of their follow up adequately described and analyzed?
11. Were participants analyzed in the groups to which they were randomized?
12. Was appropriate statistical analysis used?
13. Was the trial design appropriate and any deviations from the standard RCT design accounted for in the conduct and analysis of the trial?

Supplementary Table S6: Assessment of quality of cross-sectional studies with Joanna Briggs Institute Tools.

Author	Publication Year	Q1	Q2	Q3	Q4	Q5	Q6	Q7	Q8	Risk of Bias
Tricoli	2018	Yes	Yes	Yes	Yes	Yes	Yes	Yes	Yes	Low
Puccini	2019	Yes	Yes	Yes	Yes	No	No	Yes	Yes	Moderate
Tapial	2019	Yes	Yes	Yes	Yes	No	No	Yes	Yes	Moderate
Gao	2022	Yes	Yes	Yes	Yes	Yes	No	Yes	Yes	Low
Myer	2022	Yes	Yes	Yes	Yes	Yes	No	Yes	Yes	Low
Ugai	2022	Yes	Yes	Yes	Yes	Yes	Yes	Yes	Yes	Low
Altshuler	2023	Yes	Yes	Yes	Yes	Yes	Yes	Yes	Yes	Low
Perez-Mayoral	2023	Yes	Yes	Yes	Yes	Yes	No	Yes	Yes	Low
Ugai	2023	Yes	Yes	Yes	Yes	Yes	Yes	Yes	Yes	Low
Aruguipa	2024	Yes	Yes	Yes	Yes	Yes	No	Yes	Yes	Low
Chi	2024	Yes	Yes	Yes	Yes	Yes	Yes	Yes	Yes	Low
Kundranda	2024	Yes	Yes	Yes	Yes	Yes	No	Yes	Yes	Low
Ansar	2025	Yes	Yes	Yes	Yes	Yes	No	Yes	Yes	Low
Li	2025	Yes	Yes	Yes	Yes	Yes	Yes	Yes	Yes	Low
Okagawa	2025	Yes	Yes	Yes	Yes	Yes	No	Yes	Yes	Low
Storandt	2025	Yes	Yes	Yes	Yes	Yes	No	Yes	Yes	Low

JBI Critical Appraisal Checklist for Cross-Sectional Studies:

1) Were the criteria for inclusion in the sample clearly defined?
2) Were the study subjects and the setting described in detail?
3) Were the exposure measured in a valid and reliable way?
4) Were objective, standard criteria used for measurement of the condition?
5) Were confounding factors identified?
6) Were strategies to deal with confounding factors stated?
7) Were the outcomes measured in a valid and reliable way?
8) Was appropriate statistical analysis used?

Supplementary Table S7: Sensitivity analyses in which small studies with sample size < 100 were excluded and studies that defined EOCRC as age ≤ 50 years were excluded.

Classification	Gene	All studies				Sensitivity analysis (Excluded studies with sample size <100)				Sensitivity Analysis (Excluded studies that defined EOCRC as age ≤ 50 years)			
		No. of studies	OR (95% CI)	P-value	I^2 (%)	No. of studies	OR (95% CI)	P-value	I^2 (%)	No. of studies	OR (95% CI)	P-value	I^2 (%)
Tumour Suppressors	*TP53*	22	1.13 (1.00-1.26)	0.04	71	15	1.13 (1.01-1.27)	0.04	77	18	1.15 (1.02-1.29)	0.02	73
	APC	20	0.73 (0.61-0.89)	0.001	92	14	0.77 (0.64-0.94)	0.009	93	16	0.73 (0.59-0.91)	0.006	94
	SMAD4	20	1.15 (1.03-1.29)	0.02	35	14	1.14 (1.02-1.28)	0.02	44	17	1.15 (1.01-1.31)	0.03	45
	ATM	16	1.02 (0.85-1.23)	0.82	62	11	0.97(0.82-1.14)	0.68	58	14	1.06 (0.85-1.31)	0.61	71
	FBXW7	18	1.02 (0.95-1.09)	0.55	0	13	1.02 (0.95-1.09)	0.61	0	15	1.01 (0.94-1.08)	0.75	0
RAS-RAF-MAPK	*BRAF*	38	0.69 (0.57-0.84)	0.0002	79	31	0.71 (0.58-0.88)	0.001	82	29	0.71 (0.58-0.88)	0.001	84
	KRAS	41	0.94 (0.89-0.99)	0.01	38	32	0.94 (0.90-0.99)	0.02	44	30	0.94 (0.90-0.99)	0.02	45
	NRAS	20	0.92 (0.83-1.02)	0.10	0	16	0.91 (0.82-1.01)	0.07	0	15	0.91 (0.82-1.01)	0.08	0
PI3K/AKT	*PIK3CA*	24	1.00 (0.92-1.10)	0.92	49	18	1.00 (0.91-1.10)	0.96	56	21	1.01 (0.91-1.11)	0.87	55
	PTEN	16	1.77 (1.13-2.78)	0.01	84	12	1.75 (1.09-2.82)	0.02	88	13	2.00 (1.18-3.37)	0.01	88

Supplementary Figure S1: Assessment of publication bias and small-study effects for tumor suppressor gene mutations. Panels (a), (c), and (e) show funnel plots for *TP53*, *APC*, and *SMAD4* mutations, respectively, to evaluate potential publication bias. Panels (b), (d), and (f) show forest plots from sensitivity analyses excluding studies with sample size <100 to assess the robustness of pooled estimates.

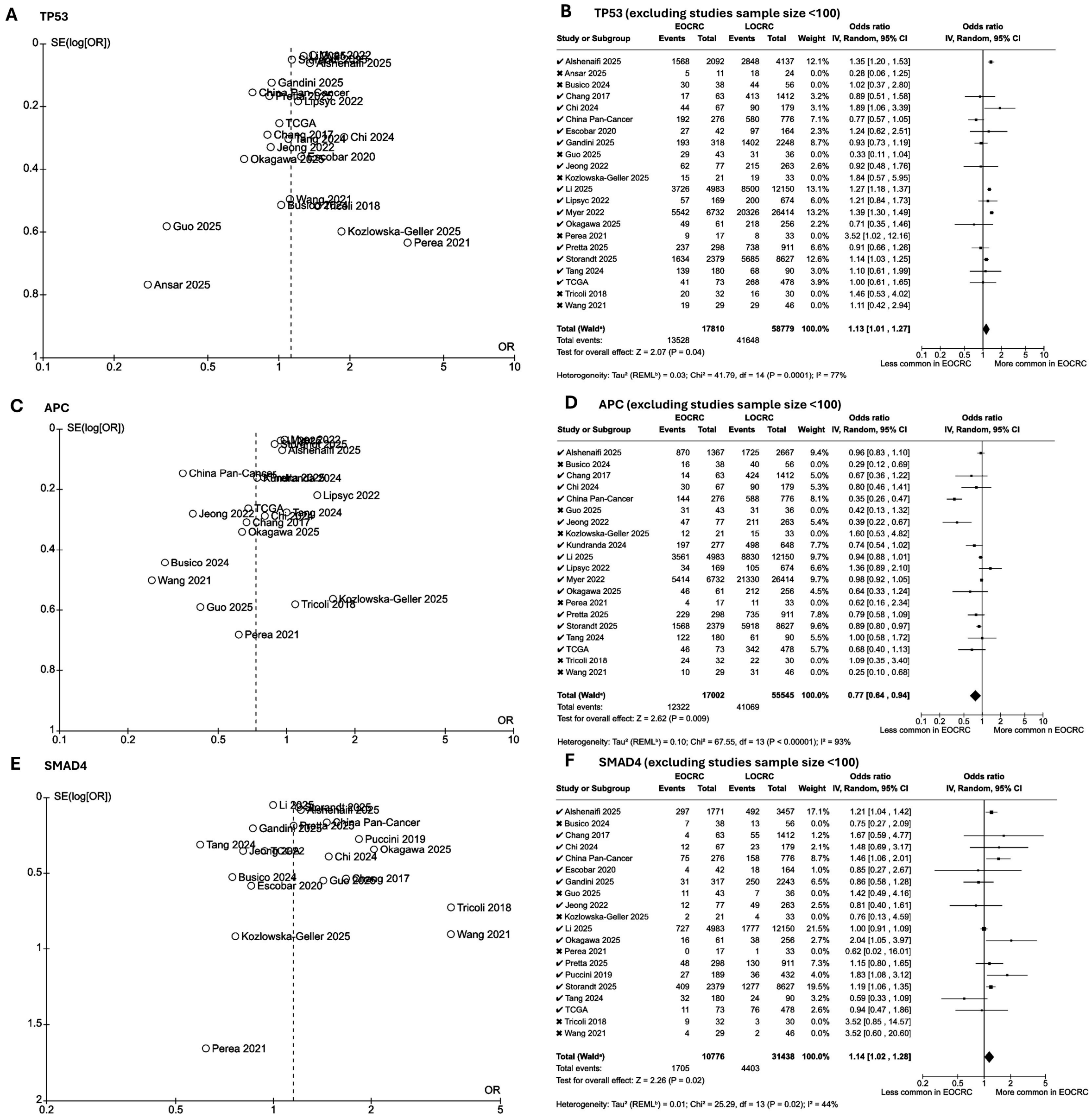

Supplementary Figure S2: Assessment of publication bias and small-study effects for RAS-RAF-MAPK pathway mutations. Panels (a), (c), and (e) show funnel plots for *BRAF*, *KRAS*, and *NRAS* mutations, respectively, to evaluate potential publication bias. Panels (b), (d), and (f) show forest plots from sensitivity analyses excluding studies with sample size <100 to assess the robustness of pooled estimates.

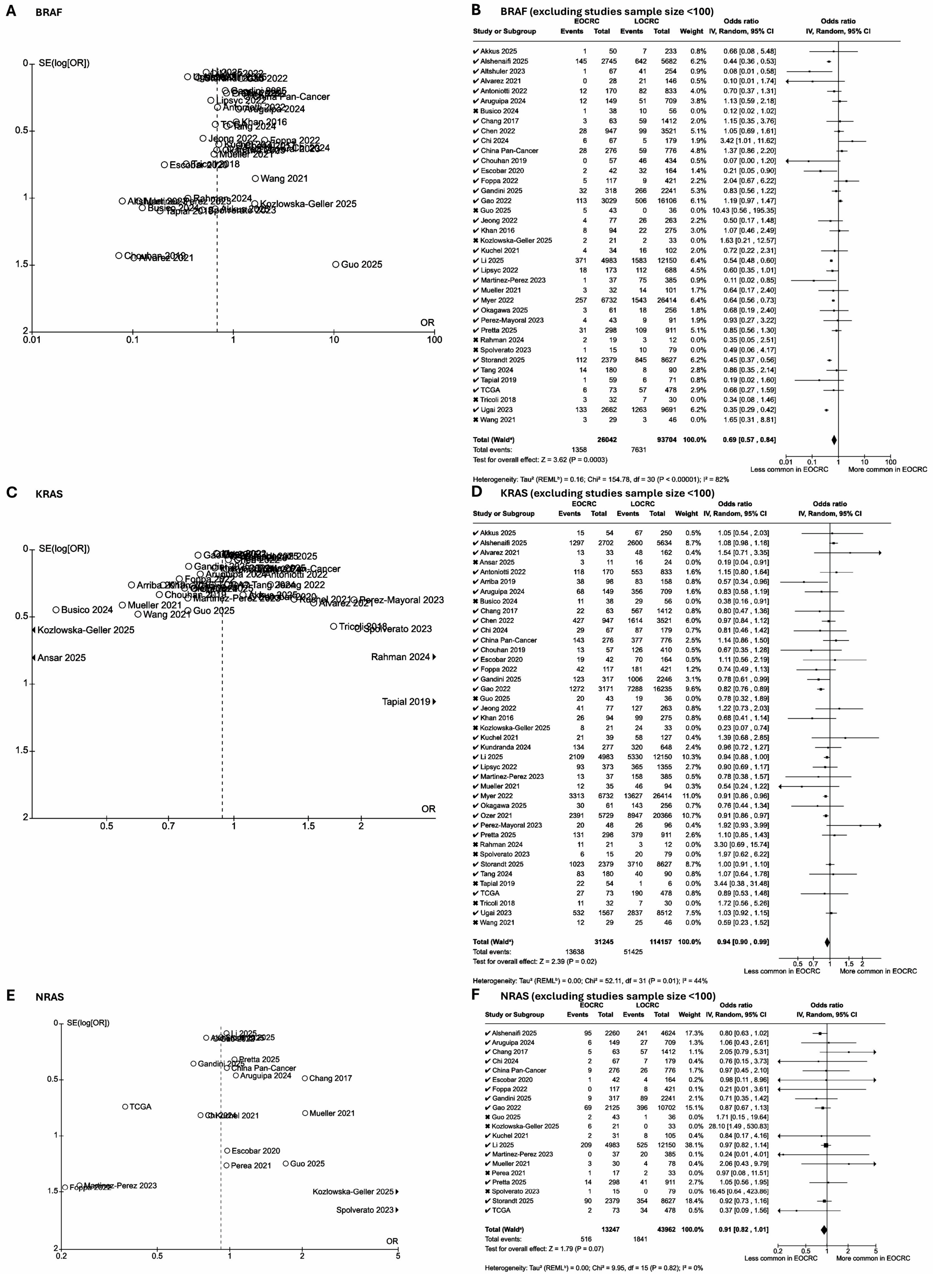

Supplementary Figure S3: Assessment of publication bias and small-study effects for PI3K/AKT pathway, *ATM* and *FBXW7* mutations. Panels (a), (c), (e), and (g) show funnel plots for *PIK3CA*, *PTEN*, *ATM*, and FBXW7 mutations, respectively, to evaluate potential publication bias. Panels (b), (d), (f), and (h) show forest plots from sensitivity analyses excluding studies with sample size <100 to assess the robustness of pooled estimates.

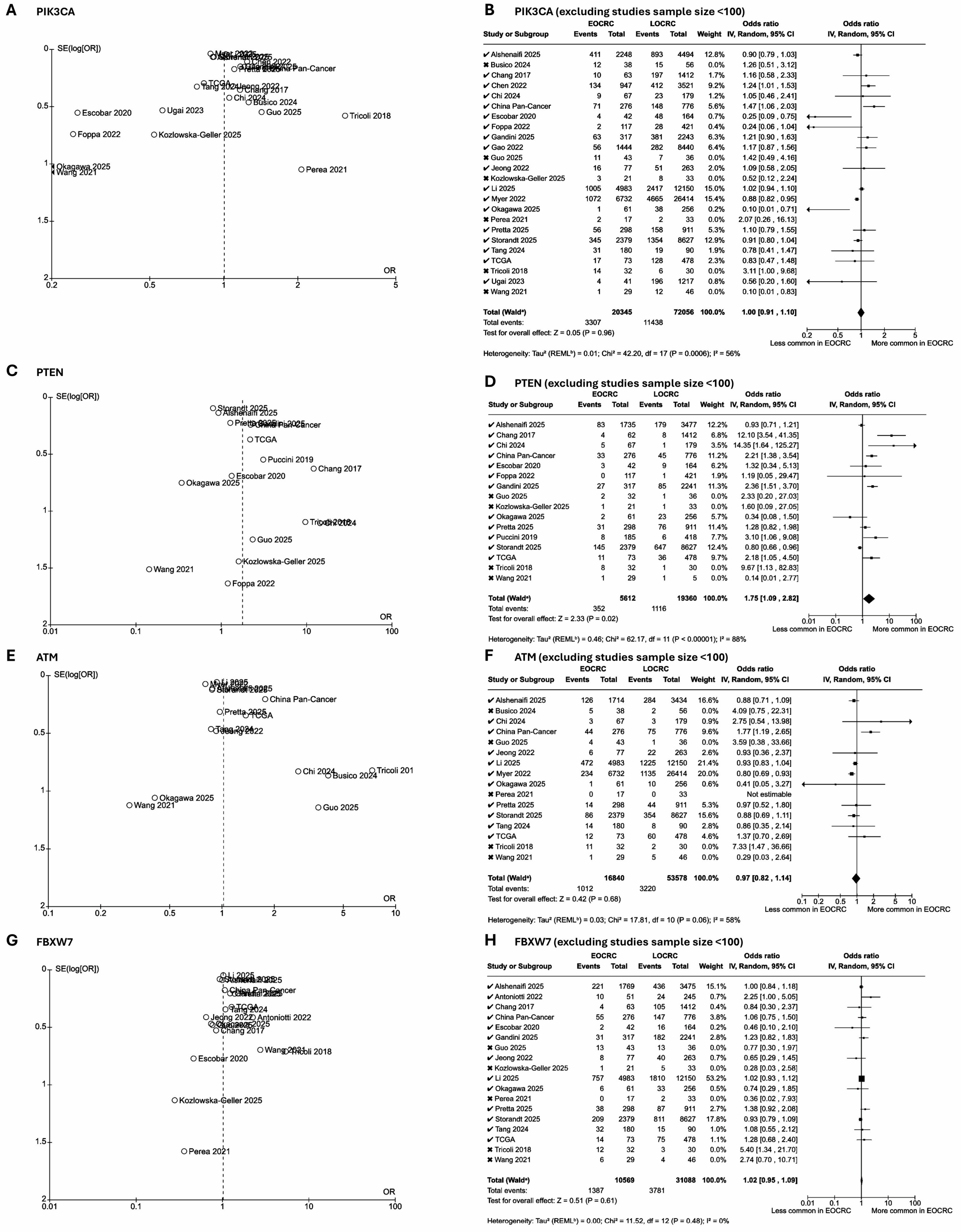

Supplementary Figure S4: Sensitivity analysis for genes *TP53, APC, SMAD4, BRAF, KRAS, NRAS* in which studies that defined EOCRC as age ≤ 50 years were excluded.

A **TP53 (Excluding studies defining EOCRC ≤50 years old)**

Study or Subgroup	EOCRC Events	EOCRC Total	LOCRC Events	LOCRC Total	Weight	Odds ratio IV, Random, 95% CI
✔ Alshenaifi 2025	1568	2092	2848	4137	12.4%	1.35 [1.20 , 1.53]
✖ Ansar 2025	5	11	18	24	0.0%	0.28 [0.06 , 1.25]
✔ Busico 2024	30	38	44	56	1.2%	1.02 [0.37 , 2.80]
✔ Chang 2017	17	63	413	1412	3.3%	0.89 [0.51 , 1.58]
✔ Chi 2024	44	67	90	179	3.2%	1.89 [1.06 , 3.39]
✔ China Pan-Cancer	192	276	580	776	7.3%	0.77 [0.57 , 1.05]
✔ Escobar 2020	27	42	97	164	2.3%	1.24 [0.62 , 2.51]
✔ Gandini 2025	193	318	1402	2248	8.9%	0.93 [0.73 , 1.19]
✔ Guo 2025	29	43	31	36	1.0%	0.33 [0.11 , 1.04]
✔ Jeong 2022	62	77	215	263	2.7%	0.92 [0.48 , 1.76]
✖ Kozlowska-Geller 2025	15	21	19	33	0.0%	1.84 [0.57 , 5.95]
✔ Li 2025	3726	4983	8500	12150	13.5%	1.27 [1.18 , 1.37]
✔ Lipsyc 2022	57	169	200	674	6.1%	1.21 [0.84 , 1.73]
✔ Myer 2022	5542	6732	20326	26414	13.6%	1.39 [1.30 , 1.49]
✔ Okagawa 2025	49	61	218	256	2.3%	0.71 [0.35 , 1.46]
✔ Perea 2021	9	17	8	33	0.8%	3.52 [1.02 , 12.16]
✖ Pretta 2025	237	298	738	911	0.0%	0.91 [0.66 , 1.26]
✔ Storandt 2025	1634	2379	5685	8627	13.0%	1.14 [1.03 , 1.25]
✔ Tang 2024	139	180	68	90	3.1%	1.10 [0.61 , 1.99]
✔ TCGA	41	73	268	478	4.0%	1.00 [0.61 , 1.65]
✔ Tricoli 2018	20	32	16	30	1.2%	1.46 [0.53 , 4.02]
✖ Wang 2021	19	29	29	46	0.0%	1.11 [0.42 , 2.94]
**Total (Wald[a])**		**17642**		**58023**	**100.0%**	**1.15 [1.02 , 1.29]**
Total events:	13379		41009			

Test for overall effect: Z = 2.29 (P = 0.02)

Heterogeneity: Tau^2 (REML[b]) = 0.03; Chi^2 = 46.02, df = 17 (P = 0.0002); I^2 = 73%

Axis: 0.1, 0.2, 0.5, 1, 2, 5, 10 — Less common in EOCRC / More common in EOCRC

B **APC (Excluding studies defining EOCRC ≤50 years old)**

Study or Subgroup	EOCRC Events	EOCRC Total	LOCRC Events	LOCRC Total	Weight	Odds ratio IV, Random, 95% CI
✔ Alshenaifi 2025	870	1367	1725	2667	9.4%	0.96 [0.83 , 1.10]
✔ Busico 2024	16	38	40	56	3.9%	0.29 [0.12 , 0.69]
✔ Chang 2017	14	63	424	1412	5.6%	0.67 [0.36 , 1.22]
✔ Chi 2024	30	67	90	179	5.9%	0.80 [0.46 , 1.41]
✔ China Pan-Cancer	144	276	588	776	8.3%	0.35 [0.26 , 0.47]
✔ Guo 2025	31	43	31	36	2.6%	0.42 [0.13 , 1.32]
✔ Jeong 2022	47	77	211	263	6.0%	0.39 [0.22 , 0.67]
✖ Kozlowska-Geller 2025	12	21	15	33	0.0%	1.60 [0.53 , 4.82]
✖ Kundranda 2024	197	277	498	648	0.0%	0.74 [0.54 , 1.02]
✔ Li 2025	3561	4983	8830	12150	9.6%	0.94 [0.88 , 1.01]
✔ Lipsyc 2022	34	169	105	674	7.1%	1.36 [0.89 , 2.10]
✔ Myer 2022	5414	6732	21330	26414	9.6%	0.98 [0.92 , 1.05]
✔ Okagawa 2025	46	61	212	256	5.1%	0.64 [0.33 , 1.24]
✔ Perea 2021	4	17	11	33	2.1%	0.62 [0.16 , 2.34]
✖ Pretta 2025	229	298	735	911	0.0%	0.79 [0.58 , 1.09]
✔ Storandt 2025	1568	2379	5918	8627	9.5%	0.89 [0.80 , 0.97]
✔ Tang 2024	122	180	61	90	6.1%	1.00 [0.58 , 1.72]
✔ TCGA	46	73	342	478	6.3%	0.68 [0.40 , 1.13]
✔ Tricoli 2018	24	32	22	30	2.7%	1.09 [0.35 , 3.40]
✖ Wang 2021	10	29	31	46	0.0%	0.25 [0.10 , 0.68]
**Total (Wald[a])**		**16557**		**54141**	**100.0%**	**0.73 [0.59 , 0.91]**
Total events:	11971		39940			

Test for overall effect: Z = 2.76 (P = 0.006)

Heterogeneity: Tau^2 (REML[b]) = 0.13; Chi^2 = 74.02, df = 15 (P < 0.00001); I^2 = 94%

Axis: 0.1, 0.2, 0.5, 1, 2, 5, 10 — Less common in EOCRC / More common n EOCRC

C **SMAD4 (Excluding studies defining EOCRC ≤50 years old)**

Study or Subgroup	EOCRC Events	EOCRC Total	LOCRC Events	LOCRC Total	Weight	Odds ratio IV, Random, 95% CI
✔ Alshenaifi 2025	297	1771	492	3457	17.1%	1.21 [1.04 , 1.42]
✔ Busico 2024	7	38	13	56	1.5%	0.75 [0.27 , 2.09]
✔ Chang 2017	4	63	55	1412	1.4%	1.67 [0.59 , 4.77]
✔ Chi 2024	12	67	23	179	2.5%	1.48 [0.69 , 3.17]
✔ China Pan-Cancer	75	276	158	776	9.5%	1.46 [1.06 , 2.01]
✔ Escobar 2020	4	42	18	164	1.2%	0.85 [0.27 , 2.67]
✔ Gandini 2025	31	317	250	2243	7.3%	0.86 [0.58 , 1.28]
✔ Guo 2025	11	43	7	36	1.3%	1.42 [0.49 , 4.16]
✔ Jeong 2022	12	77	49	263	3.0%	0.81 [0.40 , 1.61]
✖ Kozlowska-Geller 2025	2	21	4	33	0.0%	0.76 [0.13 , 4.59]
✔ Li 2025	727	4983	1777	12150	20.5%	1.00 [0.91 , 1.09]
✔ Okagawa 2025	16	61	38	256	3.2%	2.04 [1.05 , 3.97]
✔ Perea 2021	0	17	1	33	0.2%	0.62 [0.02 , 16.01]
✖ Pretta 2025	48	298	130	911	0.0%	1.15 [0.80 , 1.65]
✔ Puccini 2019	27	189	36	432	4.7%	1.83 [1.08 , 3.12]
✔ Storandt 2025	409	2379	1277	8627	19.0%	1.19 [1.06 , 1.35]
✔ Tang 2024	32	180	24	90	3.8%	0.59 [0.33 , 1.09]
✔ TCGA	11	73	76	478	3.0%	0.94 [0.47 , 1.86]
✔ Tricoli 2018	9	32	3	30	0.8%	3.52 [0.85 , 14.57]
✖ Wang 2021	4	29	2	46	0.0%	3.52 [0.60 , 20.60]
**Total (Wald[a])**		**10608**		**30682**	**100.0%**	**1.15 [1.01 , 1.31]**
Total events:	1684		4297			

Test for overall effect: Z = 2.14 (P = 0.03)

Heterogeneity: Tau^2 (REML[b]) = 0.02; Chi^2 = 28.69, df = 16 (P = 0.03); I^2 = 45%

Axis: 0.2, 0.5, 1, 2, 5 — Less common in EOCRC / More common in EOCRC

D **BRAF (Excluding studies defining EOCRC ≤50 years old)**

Study or Subgroup	EOCRC Events	EOCRC Total	LOCRC Events	LOCRC Total	Weight	Odds ratio IV, Random, 95% CI
✔ Akkus 2025	1	50	7	233	0.8%	0.66 [0.08 , 5.48]
✔ Alshenaifi 2025	145	2745	642	5682	6.6%	0.44 [0.36 , 0.53]
✖ Altshuler 2023	1	67	41	254	0.0%	0.08 [0.01 , 0.58]
✖ Alvarez 2021	0	28	21	146	0.0%	0.10 [0.01 , 1.74]
✔ Antoniotti 2022	12	170	82	833	4.2%	0.70 [0.37 , 1.31]
✔ Aruguipa 2024	12	149	51	709	4.1%	1.13 [0.59 , 2.18]
✔ Busico 2024	1	38	10	56	0.9%	0.12 [0.02 , 1.02]
✔ Chang 2017	3	63	59	1412	2.1%	1.15 [0.35 , 3.76]
✔ Chen 2022	28	947	99	3521	5.4%	1.05 [0.69 , 1.61]
✔ Chi 2024	6	67	5	179	2.0%	3.42 [1.01 , 11.62]
✔ China Pan-Cancer	28	276	59	776	5.1%	1.37 [0.86 , 2.20]
✖ Chouhan 2019	0	57	46	434	0.0%	0.07 [0.00 , 1.20]
✔ Escobar 2020	2	42	32	164	1.6%	0.21 [0.05 , 0.90]
✔ Foppa 2022	5	117	9	421	2.3%	2.04 [0.67 , 6.22]
✔ Gandini 2025	32	318	266	2241	5.6%	0.83 [0.56 , 1.22]
✔ Gao 2022	113	3029	506	16106	6.5%	1.19 [0.97 , 1.47]
✔ Guo 2025	5	43	0	36	0.5%	10.43 [0.56 , 195.35]
✔ Jeong 2022	4	77	26	263	2.4%	0.50 [0.17 , 1.48]
✔ Khan 2016	8	94	22	275	3.2%	1.07 [0.46 , 2.49]
✖ Kozlowska-Geller 2025	2	21	2	33	0.0%	1.63 [0.21 , 12.57]
✖ Kuchel 2021	4	34	16	102	0.0%	0.72 [0.22 , 2.31]
✔ Li 2025	371	4983	1583	12150	6.8%	0.54 [0.48 , 0.60]
✔ Lipsyc 2022	18	173	112	688	4.8%	0.60 [0.35 , 1.01]
✖ Martinez-Perez 2023	1	37	75	385	0.0%	0.11 [0.02 , 0.85]
✔ Mueller 2021	3	32	14	101	1.8%	0.64 [0.17 , 2.40]
✔ Myer 2022	257	6732	1543	26414	6.7%	0.64 [0.56 , 0.73]
✔ Okagawa 2025	3	61	18	256	2.0%	0.68 [0.19 , 2.40]
✔ Perez-Mayoral 2023	4	43	9	91	2.0%	0.93 [0.27 , 3.22]
✖ Pretta 2025	31	298	109	911	0.0%	0.85 [0.56 , 1.30]
✔ Rahman 2024	2	19	3	12	1.0%	0.35 [0.05 , 2.51]
✖ Spolverato 2023	1	15	10	79	0.0%	0.49 [0.06 , 4.17]
✔ Storandt 2025	112	2379	845	8627	6.5%	0.45 [0.37 , 0.56]
✔ Tang 2024	14	180	8	90	3.0%	0.86 [0.35 , 2.14]
✔ Tapial 2019	1	59	6	71	0.8%	0.19 [0.02 , 1.60]
✔ TCGA	6	73	57	478	3.1%	0.66 [0.27 , 1.59]
✔ Tricoli 2018	3	32	7	30	1.6%	0.34 [0.08 , 1.46]
✔ Ugai 2023	133	2662	1263	9691	6.6%	0.35 [0.29 , 0.42]
✖ Wang 2021	3	29	3	46	0.0%	1.65 [0.31 , 8.81]
**Total (Wald[a])**		**25653**		**91606**	**100.0%**	**0.71 [0.58 , 0.88]**
Total events:	1332		7343			

Test for overall effect: Z = 3.21 (P = 0.001)

Heterogeneity: Tau^2 (REML[b]) = 0.16; Chi^2 = 148.23, df = 28 (P < 0.00001); I^2 = 84%

Axis: 0.01, 0.1, 1, 10, 100 — Less common in EOCRC / More common in EOCRC

E **KRAS (Excluding studies defining EOCRC ≤50 years old)**

Study or Subgroup	EOCRC Events	EOCRC Total	LOCRC Events	LOCRC Total	Weight	Odds ratio IV, Random, 95% CI
✔ Akkus 2025	15	54	67	250	0.6%	1.05 [0.54 , 2.03]
✔ Alshenaifi 2025	1297	2702	2600	5634	9.3%	1.08 [0.98 , 1.18]
✖ Alvarez 2021	13	33	48	162	0.0%	1.54 [0.71 , 3.35]
✖ Ansar 2025	3	11	16	24	0.0%	0.19 [0.04 , 0.91]
✔ Antoniotti 2022	118	170	553	833	1.7%	1.15 [0.80 , 1.64]
✖ Arriba 2019	38	98	83	158	0.0%	0.57 [0.34 , 0.96]
✔ Aruguipa 2024	68	149	356	709	1.8%	0.83 [0.58 , 1.19]
✔ Busico 2024	11	38	29	56	0.3%	0.38 [0.16 , 0.91]
✔ Chang 2017	22	63	567	1412	0.8%	0.80 [0.47 , 1.36]
✔ Chen 2022	427	947	1614	3521	6.4%	0.97 [0.84 , 1.12]
✔ Chi 2024	29	67	87	179	0.7%	0.81 [0.46 , 1.42]
✔ China Pan-Cancer	143	276	377	776	2.7%	1.14 [0.86 , 1.50]
✖ Chouhan 2019	13	57	126	410	0.0%	0.67 [0.35 , 1.28]
✔ Escobar 2020	19	42	70	164	0.5%	1.11 [0.56 , 2.19]
✔ Foppa 2022	42	117	181	421	1.3%	0.74 [0.49 , 1.13]
✔ Gandini 2025	123	317	1006	2246	3.3%	0.78 [0.61 , 0.99]
✔ Gao 2022	1272	3171	7288	16235	10.3%	0.82 [0.76 , 0.89]
✔ Guo 2025	20	43	19	36	0.3%	0.78 [0.32 , 1.89]
✔ Jeong 2022	41	77	127	263	0.9%	1.22 [0.73 , 2.03]
✔ Khan 2016	26	94	99	275	0.9%	0.68 [0.41 , 1.14]
✖ Kozlowska-Geller 2025	8	21	24	33	0.0%	0.23 [0.07 , 0.74]
✖ Kuchel 2021	21	39	58	127	0.0%	1.39 [0.68 , 2.85]
✖ Kundranda 2024	134	277	320	648	0.0%	0.96 [0.72 , 1.27]
✔ Li 2025	2109	4983	5330	12150	11.0%	0.94 [0.88 , 1.00]
✔ Lipsyc 2022	93	373	365	1355	2.9%	0.90 [0.69 , 1.17]
✖ Martinez-Perez 2023	13	37	158	385	0.0%	0.78 [0.38 , 1.57]
✔ Mueller 2021	12	35	46	94	0.4%	0.54 [0.24 , 1.22]
✔ Myer 2022	3313	6732	13627	26414	11.8%	0.91 [0.86 , 0.96]
✔ Okagawa 2025	30	61	143	256	0.8%	0.76 [0.44 , 1.34]
✔ Ozer 2021	2391	5729	8947	20366	11.4%	0.91 [0.86 , 0.97]
✔ Perez-Mayoral 2023	20	48	26	96	0.5%	1.92 [0.93 , 3.99]
✖ Pretta 2025	131	298	379	911	0.0%	1.10 [0.85 , 1.43]
✔ Rahman 2024	11	21	3	12	0.1%	3.30 [0.69 , 15.74]
✖ Spolverato 2023	6	15	20	79	0.0%	1.97 [0.62 , 6.22]
✔ Storandt 2025	1023	2379	3710	8627	9.3%	1.00 [0.91 , 1.10]
✔ Tang 2024	83	180	40	90	0.9%	1.07 [0.64 , 1.78]
✔ Tapial 2019	22	54	1	6	0.1%	3.44 [0.38 , 31.48]
✔ TCGA	27	73	190	478	0.9%	0.89 [0.53 , 1.48]
✔ Tricoli 2018	11	32	7	30	0.2%	1.72 [0.56 , 5.26]
✔ Ugai 2023	532	1567	2837	8512	8.0%	1.03 [0.92 , 1.15]
✖ Wang 2021	12	29	25	46	0.0%	0.59 [0.23 , 1.52]
**Total (Wald[a])**		**30594**		**111496**	**100.0%**	**0.94 [0.90 , 0.99]**
Total events:	13350		50312			

Test for overall effect: Z = 2.36 (P = 0.02)

Heterogeneity: Tau^2 (REML[b]) = 0.00; Chi^2 = 52.21, df = 29 (P = 0.005); I^2 = 45%

Axis: 0.5, 0.7, 1, 1.5, 2 — Less common in EOCRC / More common in EOCRC

F **NRAS (Excluding studies defining EOCRC ≤50 years old)**

Study or Subgroup	EOCRC Events	EOCRC Total	LOCRC Events	LOCRC Total	Weight	Odds ratio IV, Random, 95% CI
✔ Alshenaifi 2025	95	2260	241	4624	17.8%	0.80 [0.63 , 1.02]
✔ Aruguipa 2024	6	149	27	709	1.3%	1.06 [0.43 , 2.61]
✔ Chang 2017	5	63	57	1412	1.2%	2.05 [0.79 , 5.31]
✔ Chi 2024	2	67	7	179	0.4%	0.76 [0.15 , 3.73]
✔ China Pan-Cancer	9	276	26	776	1.8%	0.97 [0.45 , 2.10]
✔ Escobar 2020	1	42	4	164	0.2%	0.98 [0.11 , 8.96]
✔ Foppa 2022	0	117	8	421	0.1%	0.21 [0.01 , 3.61]
✔ Gandini 2025	9	317	89	2241	2.2%	0.71 [0.35 , 1.42]
✔ Gao 2022	69	2125	396	10702	15.6%	0.87 [0.67 , 1.13]
✔ Guo 2025	2	43	1	36	0.2%	1.71 [0.15 , 19.64]
✖ Kozlowska-Geller 2025	6	21	0	33	0.0%	28.10 [1.49 , 530.83]
✖ Kuchel 2021	2	31	8	105	0.0%	0.84 [0.17 , 4.16]
✔ Li 2025	209	4983	525	12150	39.2%	0.97 [0.82 , 1.14]
✖ Martinez-Perez 2023	0	37	20	385	0.0%	0.24 [0.01 , 4.01]
✔ Mueller 2021	3	30	4	78	0.4%	2.06 [0.43 , 9.79]
✔ Perea 2021	1	17	2	33	0.2%	0.97 [0.08 , 11.51]
✖ Pretta 2025	14	298	41	911	0.0%	1.05 [0.56 , 1.95]
✖ Spolverato 2023	1	15	0	79	0.0%	16.45 [0.64 , 423.86]
✔ Storandt 2025	90	2379	354	8627	18.9%	0.92 [0.73 , 1.16]
✔ TCGA	2	73	34	478	0.5%	0.37 [0.09 , 1.56]
**Total (Wald[a])**		**12941**		**42630**	**100.0%**	**0.91 [0.82 , 1.01]**
Total events:	503		1775			

Test for overall effect: Z = 1.77 (P = 0.08)

Heterogeneity: Tau^2 (REML[b]) = 0.00; Chi^2 = 9.13, df = 14 (P = 0.82); I^2 = 0%

Axis: 0.2, 0.5, 1, 2, 5 — Less common in EOCRC / More common in EOCRC

Footnotes

[a]CI calculated by Wald-type method.

[b]Tau^2 calculated by Restricted Maximum-Likelihood method.

Supplementary Figure S5: Sensitivity analysis for genes *PIK3CA*, *PTEN*, *ATM* and *FBXW7* in which studies that defined EOCRC as age ≤ 50 years were excluded.

A PIK3CA (Excluding studies defining EOCRC ≤50 years old)

Study or Subgroup	EOCRC Events	EOCRC Total	LOCRC Events	LOCRC Total	Weight	Odds ratio IV, Random, 95% CI
✔ Alshenaifi 2025	411	2248	893	4494	12.9%	0.90 [0.79 , 1.03]
✔ Busico 2024	12	38	15	56	1.1%	1.26 [0.51 , 3.12]
✔ Chang 2017	10	63	197	1412	1.8%	1.16 [0.58 , 2.33]
✔ Chen 2022	134	947	412	3521	9.5%	1.24 [1.01 , 1.53]
✔ Chi 2024	9	67	23	179	1.3%	1.05 [0.46 , 2.41]
✔ China Pan-Cancer	71	276	148	776	6.0%	1.47 [1.06 , 2.03]
✔ Escobar 2020	4	42	48	164	0.8%	0.25 [0.09 , 0.75]
✔ Foppa 2022	2	117	28	421	0.5%	0.24 [0.06 , 1.04]
✔ Gandini 2025	63	317	381	2243	6.7%	1.21 [0.90 , 1.63]
✔ Gao 2022	56	1444	282	8440	6.8%	1.17 [0.87 , 1.56]
✔ Guo 2025	11	43	7	36	0.8%	1.42 [0.49 , 4.16]
✔ Jeong 2022	16	77	51	263	2.2%	1.09 [0.58 , 2.05]
✖ Kozlowska-Geller 2025	3	21	8	33	0.0%	0.52 [0.12 , 2.24]
✔ Li 2025	1005	4983	2417	12150	14.8%	1.02 [0.94 , 1.10]
✔ Myer 2022	1072	6732	4665	26414	15.1%	0.88 [0.82 , 0.95]
✔ Okagawa 2025	1	61	38	256	0.2%	0.10 [0.01 , 0.71]
✔ Perea 2021	2	17	2	33	0.2%	2.07 [0.26 , 16.13]
✖ Pretta 2025	56	298	158	911	0.0%	1.10 [0.79 , 1.55]
✔ Storandt 2025	345	2379	1354	8627	12.9%	0.91 [0.80 , 1.04]
✔ Tang 2024	31	180	19	90	2.1%	0.78 [0.41 , 1.47]
✔ TCGA	17	73	128	478	2.5%	0.83 [0.47 , 1.48]
✔ Tricoli 2018	14	32	6	30	0.7%	3.11 [1.00 , 9.68]
✔ Ugai 2023	4	41	196	1217	0.9%	0.56 [0.20 , 1.60]
✖ Wang 2021	1	29	12	46	0.0%	0.10 [0.01 , 0.83]
**Total (Wald[a])**		**20177**		**71300**	**100.0%**	**1.01 [0.91 , 1.11]**
Total events:	3290		11310			

Test for overall effect: Z = 0.17 (P = 0.87)

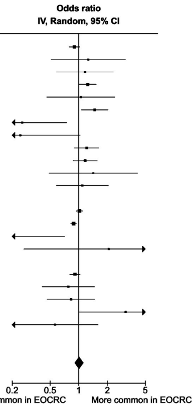

Heterogeneity: Tau^2 (REML[b]) = 0.02; Chi^2 = 47.09, df = 20 (P = 0.0006); I^2 = 55%

B PTEN (Excluding studies defining EOCRC ≤50 years old)

Study or Subgroup	EOCRC Events	EOCRC Total	LOCRC Events	LOCRC Total	Weight	Odds ratio IV, Random, 95% CI
✔ Alshenaifi 2025	83	1735	179	3477	12.2%	0.93 [0.71 , 1.21]
✔ Chang 2017	4	62	8	1412	7.4%	12.10 [3.54 , 41.35]
✔ Chi 2024	5	67	1	179	4.0%	14.35 [1.64 , 125.27]
✔ China Pan-Cancer	33	276	45	776	11.4%	2.21 [1.38 , 3.54]
✔ Escobar 2020	3	42	9	164	6.8%	1.32 [0.34 , 5.13]
✔ Foppa 2022	0	117	1	421	2.2%	1.19 [0.05 , 29.47]
✔ Gandini 2025	27	317	85	2241	11.5%	2.36 [1.51 , 3.70]
✔ Guo 2025	2	32	1	36	3.4%	2.33 [0.20 , 27.03]
✖ Kozlowska-Geller 2025	1	21	1	33	0.0%	1.60 [0.09 , 27.05]
✔ Okagawa 2025	2	61	23	256	6.3%	0.34 [0.08 , 1.50]
✖ Pretta 2025	31	298	76	911	0.0%	1.28 [0.82 , 1.98]
✔ Puccini 2019	8	185	6	418	8.2%	3.10 [1.06 , 9.08]
✔ Storandt 2025	145	2379	647	8627	12.4%	0.80 [0.66 , 0.96]
✔ TCGA	11	73	36	478	10.1%	2.18 [1.05 , 4.50]
✔ Tricoli 2018	8	32	1	30	4.0%	9.67 [1.13 , 82.83]
✖ Wang 2021	1	29	1	5	0.0%	0.14 [0.01 , 2.77]
**Total (Wald[a])**		**5378**		**18515**	**100.0%**	**2.00 [1.18 , 3.37]**
Total events:	331		1042			

Test for overall effect: Z = 2.59 (P = 0.010)

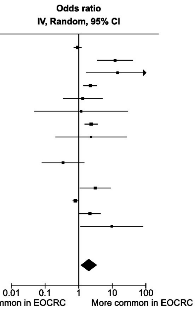

Heterogeneity: Tau^2 (REML[b]) = 0.57; Chi^2 = 66.03, df = 12 (P < 0.00001); I^2 = 88%

C ATM (Excluding studies defining EOCRC ≤50 years old)

Study or Subgroup	EOCRC Events	EOCRC Total	LOCRC Events	LOCRC Total	Weight	Odds ratio IV, Random, 95% CI
✔ Alshenaifi 2025	126	1714	284	3434	16.0%	0.88 [0.71 , 1.09]
✔ Busico 2024	5	38	2	56	1.5%	4.09 [0.75 , 22.31]
✔ Chi 2024	3	67	3	179	1.6%	2.75 [0.54 , 13.98]
✔ China Pan-Cancer	44	276	75	776	11.4%	1.77 [1.19 , 2.65]
✔ Guo 2025	4	43	1	36	0.9%	3.59 [0.38 , 33.66]
✔ Jeong 2022	6	77	22	263	4.0%	0.93 [0.36 , 2.37]
✔ Li 2025	472	4983	1225	12150	18.2%	0.93 [0.83 , 1.04]
✔ Myer 2022	234	6732	1135	26414	17.6%	0.80 [0.69 , 0.93]
✔ Okagawa 2025	1	61	10	256	1.0%	0.41 [0.05 , 3.27]
✔ Perea 2021	0	17	0	33		Not estimable
✖ Pretta 2025	14	298	44	911	0.0%	0.97 [0.52 , 1.80]
✔ Storandt 2025	86	2379	354	8627	15.4%	0.88 [0.69 , 1.11]
✔ Tang 2024	14	180	8	90	4.3%	0.86 [0.35 , 2.14]
✔ TCGA	12	73	60	478	6.6%	1.37 [0.70 , 2.69]
✔ Tricoli 2018	11	32	2	30	1.6%	7.33 [1.47 , 36.66]
✖ Wang 2021	1	29	5	46	0.0%	0.29 [0.03 , 2.64]
**Total (Wald[a])**		**16672**		**52822**	**100.0%**	**1.06 [0.85 , 1.31]**
Total events:	1018		3181			

Test for overall effect: Z = 0.51 (P = 0.61)

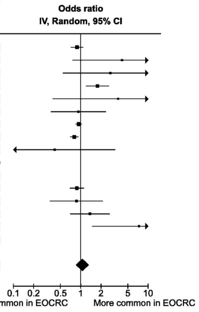

Heterogeneity: Tau^2 (REML[b]) = 0.06; Chi^2 = 28.65, df = 12 (P = 0.004); I^2 = 71%

D FBXW7 (Excluding studies defining EOCRC ≤50 years old)

Study or Subgroup	EOCRC Events	EOCRC Total	LOCRC Events	LOCRC Total	Weight	Odds ratio IV, Random, 95% CI
✔ Alshenaifi 2025	221	1769	436	3475	15.4%	1.00 [0.84 , 1.18]
✔ Antoniotti 2022	10	51	24	245	0.7%	2.25 [1.00 , 5.05]
✔ Chang 2017	4	63	105	1412	0.4%	0.84 [0.30 , 2.37]
✔ China Pan-Cancer	55	276	147	776	3.8%	1.06 [0.75 , 1.50]
✔ Escobar 2020	2	42	16	164	0.2%	0.46 [0.10 , 2.10]
✔ Gandini 2025	31	317	182	2241	2.9%	1.23 [0.82 , 1.83]
✔ Guo 2025	13	43	13	36	0.5%	0.77 [0.30 , 1.97]
✔ Jeong 2022	8	77	40	263	0.7%	0.65 [0.29 , 1.45]
✖ Kozlowska-Geller 2025	1	21	5	33	0.0%	0.28 [0.03 , 2.58]
✔ Li 2025	757	4983	1810	12150	54.2%	1.02 [0.93 , 1.12]
✔ Okagawa 2025	6	61	33	256	0.5%	0.74 [0.29 , 1.85]
✔ Perea 2021	0	17	2	33	0.0%	0.36 [0.02 , 7.93]
✖ Pretta 2025	38	298	87	911	0.0%	1.38 [0.92 , 2.08]
✔ Storandt 2025	209	2379	811	8627	18.1%	0.93 [0.79 , 1.09]
✔ Tang 2024	32	180	15	90	1.0%	1.08 [0.55 , 2.12]
✔ TCGA	14	73	75	478	1.1%	1.28 [0.68 , 2.40]
✔ Tricoli 2018	12	32	3	30	0.2%	5.40 [1.34 , 21.70]
✖ Wang 2021	6	29	4	46	0.0%	2.74 [0.70 , 10.71]
**Total (Wald[a])**		**10363**		**30276**	**100.0%**	**1.01 [0.94 , 1.08]**
Total events:	1374		3712			

Test for overall effect: Z = 0.31 (P = 0.75)

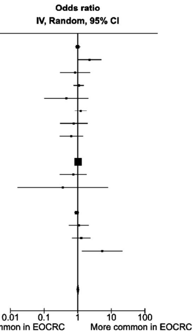

Heterogeneity: Tau^2 (REML[b]) = 0.00; Chi^2 = 15.59, df = 14 (P = 0.34); I^2 = 0%

Footnotes

[a]CI calculated by Wald-type method.

[b]Tau^2 calculated by Restricted Maximum-Likelihood method.
